# MYC inhibition by Omomyc causes DNA damage and overcomes PARPi resistance in breast cancer

**DOI:** 10.1016/j.celrep.2025.116604

**Published:** 2025-11-21

**Authors:** Fabio Giuntini, Íñigo González-Larreategui, Andrea Herencia-Ropero, Silvia Casacuberta-Serra, Mariano F. Zacarías-Fluck, Magdalena Arnal, Flaminia Pedretti, Sandra Martínez-Martín, Hugo Thabussot, Virginia Castillo Cano, Judit Grueso, Laia Foradada, Erika Serrano, Sergio López-Estévez, Olga Rodriguez, Marta Guzman, Adela Rodriguez-Hernandez, Fara Brasó-Maristany, Alba Llop-Guevara, Judith Balmaña, Lara Nonell, Aleix Prat, Violeta Serra, Marie-Eve Beaulieu, Jonathan R. Whitfield, Daniel Massó-Vallés, Laura Soucek

**Affiliations:** 1 Models of Cancer Therapies Group, Vall d’Hebron Institute of Oncology (VHIO), Vall d’Hebron Barcelona Hospital Campus, Barcelona, Spain; 2 Experimental Therapeutics Group, Vall d’Hebron Institute of Oncology (VHIO), Vall d’Hebron Barcelona Hospital Campus, Barcelona, Spain; 3 Peptomyc S.L., Vall d’Hebron Barcelona Hospital Campus, Barcelona, Spain; 4 Translational Genomics and Targeted Therapeutics in Solid Tumors, IDIBAPS, Barcelona, Spain; 5 Hereditary Cancer Genetics Group, Vall d’Hebron Institute of Oncology (VHIO), Vall d’Hebron Barcelona Hospital Campus, Barcelona, Spain; 6 Bioinformatics Unit, Vall d’Hebron Institute of Oncology (VHIO), Vall d’Hebron Barcelona Hospital Campus, Barcelona, Spain; 7 Institució Catalana de Recerca i Estudis Avançats (ICREA), Barcelona, Spain; 8 Department of Biochemistry and Molecular Biology, Universitat Autònoma de Barcelona, Bellaterra, Spain

**Keywords:** Omomyc, TNBC, PARPi, DNA damage, therapy resistance

## Abstract

MYC is dysregulated in most human cancers and is a DNA damage response (DDR) modulator capable of both promoting genomic instability and enhancing DNA repair. Here, we show that Omomyc, the only direct MYC inhibitor that has completed a phase 1 trial, shuts down DDR genes in triple-negative breast cancer (TNBC), causing DDR defects and inducing DNA damage. Since DDR-deficient tumors are currently targeted by poly ADP-ribose polymerase inhibitors (PARPis), we tested combinations with Omomyc. We show that Omomyc-induced DNA damage is enhanced by PARPis and that the inhibitors cooperate even in models with intrinsic or acquired PARPi resistance, both *in vitro* and *in vivo*. Moreover, using patient-derived models and clinical samples, we reveal a role for MYC as a predictor of PARPi resistance. Overall, our research highlights the opportunity of combining MYC inhibition by Omomyc with PARPis in PARPi-resistant TNBC, where MYC transcriptional activity represents a predictive biomarker of resistance to therapy.

## Highlights

• Omomyc disrupts DNA damage response pathways and induces DNA damage in TNBC
• MYC inhibition synergizes with PARPis in PARPi-resistant TNBC tumor models
• Elevated MYC signaling serves as a predictive biomarker for PARPi resistance
• Combined PARP and MYC targeting overcomes PARPi resistance across multiple TNBC models

## Graphical abstract

**Figure:**
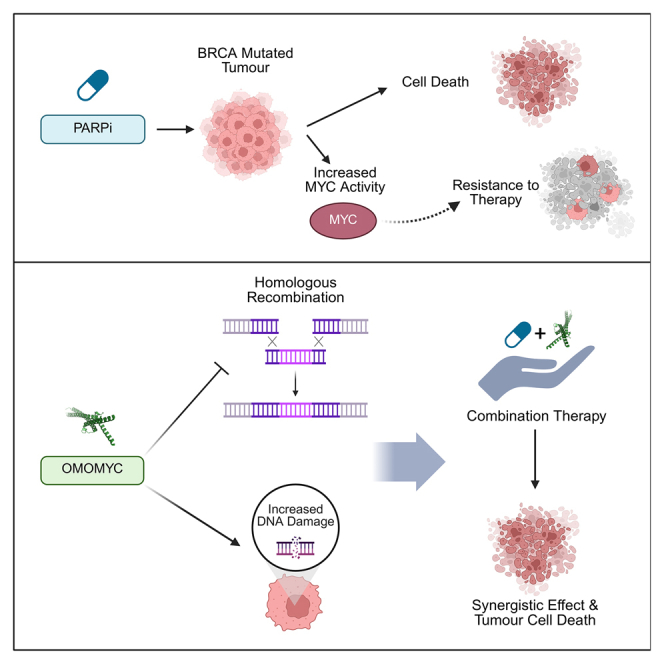

## Teaser

Giuntini et al. demonstrate that Omomyc, the only direct MYC inhibitor currently in phase 2 clinical trials, causes DNA damage and synergizes with PARPis in triple-negative breast cancer. Their findings provide compelling evidence for clinical exploration of this combination to overcome PARPi resistance.

## Introduction

MYC is a pleiotropic transcription factor, capable of regulating multiple cellular processes^1^ and found to be deregulated or overexpressed in at least 70% of cancers, where it provides proliferative and survival advantages.^2^ The oncogenic capabilities of MYC to impinge on several hallmarks of cancer, such as proliferation, modulation of the tumor microenvironment, and migration, have been well known for a few decades^3^ and have made MYC a prime target in oncology. More controversial, though, is its role within DNA damage and the DNA damage response (DDR),^4^ where MYC holds a dual nature, simultaneously promoting genomic instability through replication stress^5^ and enhancing DNA repair mechanisms via transcriptional activation of DDR genes.^6^ Such opposing functions create a delicate balance that enables cancer cells to sustain elevated levels of genomic stress while maintaining survival.^7^

Over the years, many strategies have been applied to inhibit MYC both directly and indirectly, but it has proved challenging due to its intrinsically disordered nature and lack of a small-molecule binding pocket.^8^ Because of that, until recently, no direct MYC inhibitor had appeared to be clinically viable. In this context, our group has contributed to the characterization and validation of the anti-tumorigenic potential of Omomyc, a first-in-modality MYC inhibitor, in models of multiple types of cancer^9^^,^^10^^,^^11^^,^^12^^,^^13^^,^^14^^,^^15^ and brought OMO-103 (a recombinantly produced Omomyc mini-protein) to clinical trials. A first-in-human phase 1a study was successfully completed in solid tumors in 2022,^16^ and phase 1b and phase 2 trials are currently ongoing in metastatic pancreatic cancer and advanced osteosarcoma, respectively (ClinicalTrials.gov: NCT06059001 and NCT06650514).

We previously demonstrated, at the preclinical level, the potent effect of Omomyc monotherapy in metastatic breast cancer and particularly in triple-negative breast cancer (TNBC),^11^ an aggressive subtype where MYC is disproportionally elevated as compared to other breast cancer molecular subtypes.^17^ This tumor type has few available treatments besides conventional and highly toxic chemotherapy.^18^ Immune checkpoint inhibitors such as atezolizumab and pembrolizumab have shown efficacy in combination with chemotherapy for PD-L1-positive (+) disease,^19^ while antibody-drug conjugates, including sacituzumab govitecan (targeting Trop-2)^20^ and fam-trastuzumab deruxtecan, have recently provided new therapeutic avenues for human epidermal growth factor receptor 2 (HER2)-low tumors.^21^

Another well-renown option is poly ADP-ribose polymerase (PARP) inhibitors (PARPis), which are used against tumors with a germline mutation in breast cancer type 1 susceptibility protein (*BRCA1*) and/or *BRCA2*.^22^ A minority of patients with TNBC (10%–15%) have this mutation, and, among them, many respond to PARPis only during a limited period of time due to the emergence of resistance.^23^ We report here that, during our characterization of the molecular mechanism of action underlying Omomyc’s therapeutic impact in TNBC, we observed a clear decrease in the expression of DDR-related genes, which prompted us to test the hypothesis that Omomyc treatment could induce DNA damage. In this study, we indeed show that Omomyc induces histone H2AX phosphorylation (γH2AX), both *in vitro* and *in vivo*, in TNBC models. γH2AX is a critical early molecular marker of double-strand breaks (DSBs) that serves as a rapid and sensitive mechanism to detect DNA damage.^24^ Importantly, Omomyc also triggers DNA damage in a panel of PARPi-resistant cell lines. This observation encouraged us to combine the Omomyc mini-protein and PARPi, revealing a powerful cooperative effect both *in vitro* and *in vivo*, resulting in anti-tumor response, increased DNA damage, and apoptosis. Strikingly, the combination *in vivo* in cell-line-derived and patient-derived xenografts (CDXs and PDXs, respectively) gives a synergistic therapeutic impact, achieving a higher disease control rate (DCR) than either monotherapy alone. Notably, PARPi-resistant PDXs show a significantly higher MYC transcriptional activity compared to sensitive ones, and their resistance to PARPis is overcome by Omomyc treatment. Finally, using digital spatial profiling (DSP) on patient samples, we describe the role of MYC as a predictor of clinical response to PARPis. Indeed, pre-treatment biopsies of patients with increased MYC and DDR signatures are associated with poor response to PARPis and could potentially benefit from Omomyc treatment.

Altogether, we present evidence for the central role of MYC within PARPi resistance, and we propose Omomyc as a DNA-damaging agent, capable of cooperating with PARPis and re-sensitizing PARPi-resistant TNBC to therapy.

## Results

### MYC inhibition by Omomyc downregulates DDR genes and induces DNA damage in TNBC cells

**Figure 1:**
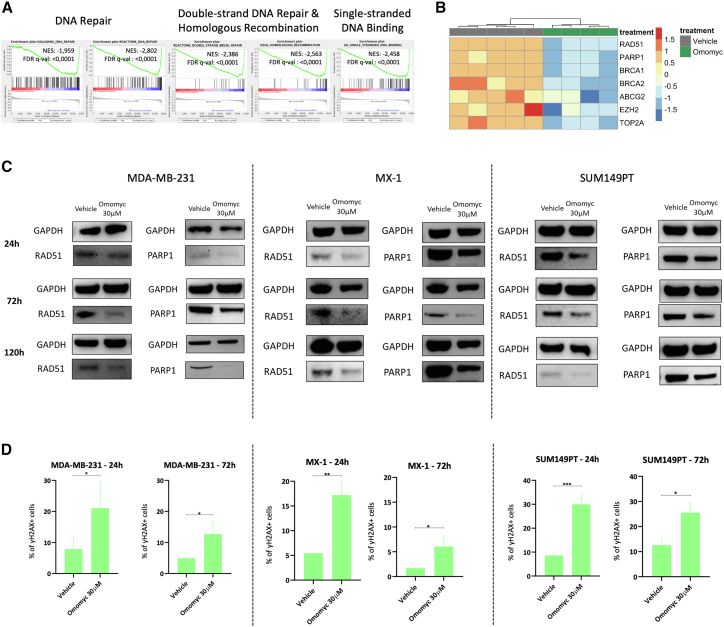
Omomyc shuts down DDR pathways and induces DNA damage (A) Microarray analysis of a panel of DDR-related gene sets treated with Omomyc mini-protein for 72 h in MDA-MB-231 cells as compared to vehicle-treated cells. (B) Microarray analysis of specific genes related to DDR in MDA-MB-231 cells treated with Omomyc for 72 h as compared to vehicle. (C) Western blot analysis of RAD51 and PARP1 protein in MDA-MB-231 (left), MX-1 (middle), and SUM149 (right) cells at 24 (top), 72 (middle), and 120 (bottom) h upon treatment with 30 μM Omomyc compared to vehicle. GAPDH was used as a loading control for each comparison. (D) Flow cytometry assessment of γH2AX+ cells when treating them for 24 or 72 h with 30 μM Omomyc as compared to vehicle-treated cells. From left to right: MDA-MB-231, MX-1, and SUM149PT cells are represented. Asterisks represent statistical significance of two-tailed *t* tests performed with GraphPad PRISM 9. ^∗^*p* < 0.05, ^∗∗^*p* < 0.01, and ^∗∗∗^*p* < 0.001; error bars, mean ± SD.

In Massó-Vallés et al.,^11^ we previously performed microarray analysis on Omomyc-treated MDA-MB-231, a TNBC benchmark cell line, and demonstrated that Omomyc treatment induces a strong shutdown of MYC transcriptional activity. Here, further analysis of the data revealed a strong correlation between MYC activity and the DDR, which was significantly affected by Omomyc treatment. Indeed, we observed the downregulation of gene sets related to DNA repair, DSB repair, single-stranded DNA binding, and homologous recombination (HR) upon Omomyc treatment for 72 h (Figure 1A). These gene sets included key effectors with statistically significant differential expression, such as *RAD51*, *PARP1*, *BRCA1*, *BRCA2*, ATP-binding cassette superfamily G member 2 (*ABCG2*), *EZH2*, and topoisomerase IIα (*TOP2A*) (Figure 1B). The downregulation of DNA repair mechanism gene sets, as well as the decreased expression of the aforementioned driver genes, was further confirmed in independent RNA sequencing (Figures S1A and S1B).

To explore the relevance of inhibiting MYC in a PARPi-resistant context, we made use of two TNBC cell lines resistant to PARPi, MX-1 and SUM149PT, and included SUM1315MO2 as a control for the PARPi response.

We first confirmed that MDA-MB-231 (BRCA wild type), MX-1, and SUM149PT (BRCA1 mutated) are resistant to both olaparib and talazoparib by calculating their 50% growth inhibition (GI 50) values in AlamarBlue proliferation assays (Figures S2A–S2C). We also verified that these three cell lines have a RAD51 and a BRCA1 C terminus (critical for BRCA1 function) scores above 10% in geminin+ cells, a marker of S-G2 phases of the cell cycle, and confirmed at least 10% HR proficiency (Figure S2D and S2E).^25^^,^^26^

Then, to test if Omomyc can reduce DDR-related genes also in this PARPi-resistant context, we treated the cells with 30 μM of the mini-protein at different time points. Downregulation of both RAD51 and PARP1 was observed in all TNBC PARPi-resistant cell lines at 24, 72, and 120 h (Figure 1C), independently of BRCA status.

To assess if these changes are related to the cell cycle, we performed cell cycle analysis at 24, 72, and 120 h in all cell lines. At these time points, Omomyc induced only a moderate arrest in G1 in MDA-MB-231 at 24 and 72 h, as previously shown in Massó-Vallés et al.,^11^ and in SUM149PT at 120 h. Overall, this suggests that the DDR changes we observe occur independently of any significant cell-cycle arrest (Figure S1C).

Given the consistent downregulation of DDR genes by Omomyc, we decided to test whether the mini-protein was able to cause DNA damage by itself. To our surprise, we observed that Omomyc was able to induce DNA damage at both 24 and 72 h in all cell lines, as demonstrated through flow cytometry by the increased percentage of cells positive for γH2AX as compared to their respective vehicle-treated cells (Figure 1D).

### Omomyc synergizes with PARPis by reducing TNBC cell viability, enhancing DNA damage, and promoting apoptosis *in vitro*

**Figure 2:**
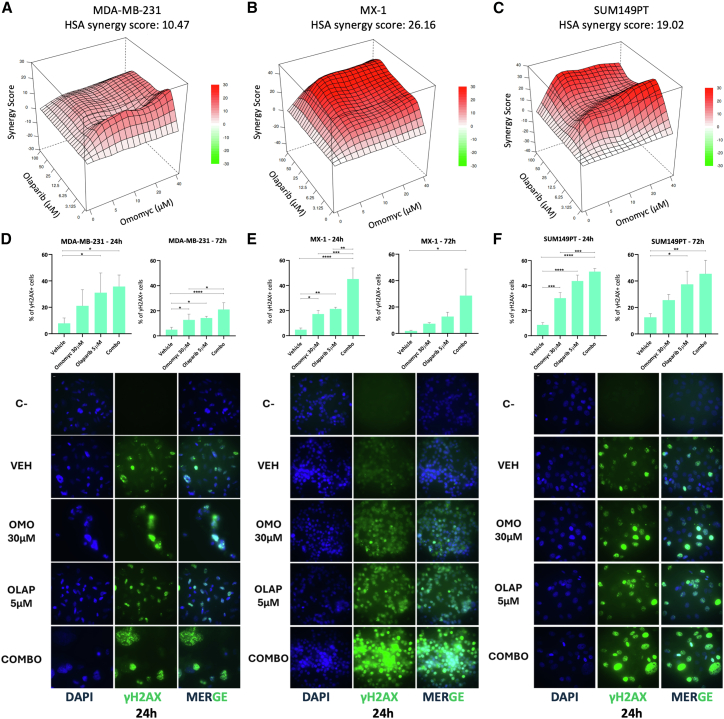
Omomyc synergizes with olaparib and enhances DNA damage in PARPi-resistant TNBC cells (A–C) Synergy plots depicting Gaddum’s non-interaction model (HSA) for MDA-MB-231 (A), MX-1 (B), and SUM149PT (C) cells treated with increasing concentrations of Omomyc and/or olaparib. (D–F) Flow cytometry analysis of γH2AX (%, *y* axis) after treatment with selected concentrations of Omomyc, olaparib, or their combination at 24 (left) or 72 (right) h in MDA-MB-231 (D), MX-1 (E), and SUM149PT (F) cells. Asterisks represent statistical significance by ordinary one-way ANOVA test with Tukey’s multiple comparisons test performed with GraphPad PRISM 9. ^∗^*p* < 0.05, ^∗∗^*p* < 0.01, and ^∗∗∗^*p* < 0.001; error bars, mean ± SD. Below the bar charts are representative fluorescent microscopy photos to show the difference observed at 24 h in each cell line. Scale, 10 μm.

In order to test whether the combination with Omomyc and PARPis could be therapeutically advantageous in a PARPi-resistant context, TNBC cells were treated with increasing concentrations of Omomyc and PARPi in a synergy matrix standard assay. All cell lines showed multiple synergistic combination doses when testing Omomyc with both olaparib (Figures 2A–2C and S3A–S3C) and talazoparib (Figures S3D–S3I). Based on the matrix, we selected 30 μM Omomyc and 5 μM olaparib to measure the levels of γH2AX by flow cytometry at 24 and 72 h after treatment (Figures 2D–2F). Omomyc and olaparib monotherapies increased γH2AX in some cell lines and time points, but their combination showed a more potent and significant increase at both time points in all cell lines. Representative images of fluorescent microscopy at 24 h are shown in Figures 2D–2F.

**Figure 3:**
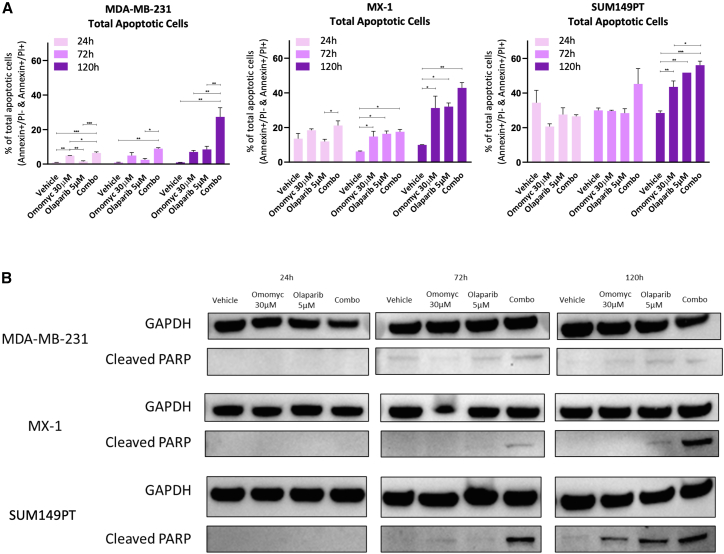
Combination of Omomyc and olaparib promotes apoptosis in PARPi-resistant TNBC cells (A) Bar charts representing total apoptosis (% of PI−/Annexin+ cells and PI+/Annexin+ cells, *y* axis) in MDA-MB-231, MX-1, and SUM149PT cells at different time points (from left to right: 24, 72, and 120 h). Treatments are indicated on the *x* axis. Asterisks represent statistical significance using an ordinary one-way ANOVA test with Tukey’s multiple comparisons test performed with GraphPad PRISM 9. ^∗^*p* < 0.05, ^∗∗^*p* < 0.01, and ^∗∗∗^*p* < 0.001; error bars, mean ± SD. (B) Western blot analysis of cleaved PARP protein in MDA-MB-231, MX-1, and SUM149PT cells at 24, 72, and 120 h upon treatment with vehicle, 30 μM Omomyc, 5 μM olaparib, or their combination. GAPDH was used as a loading control for each comparison.

Since persistent γH2AX accumulation contributes to the activation of DNA fragmentation and pro-apoptotic cascades,^27^^,^^28^ we also analyzed Omomyc’s potential role in programmed cell death within TNBC. We previously showed that transgenically expressed Omomyc was able to exacerbate MYC-induced apoptosis in high-MYC-expressing cells.^29^ Now, to assess the impact of Omomyc (30 μM), olaparib (5 μM), and their combination on apoptosis, Annexin V/PI staining was performed in MDA-MB-231, MX-1, and SUM149PT cell lines at 24, 72, and 120 h post-treatment. The total percentage of apoptotic cells (early + late apoptosis) was quantified. At 24 h, changes in apoptosis were only detected in MDA-MB-231-treated cells and below 10% of total cells, ruling out apoptosis as the cause of the dramatic γH2AX expression observed at this time point (Figures 2D–2F). By 72 h, a modest increase in apoptosis was detected only in the combination group compared to controls and monotherapies. At 120 h, on the other hand, both Omomyc and olaparib as monotherapies resulted in a clear increase in apoptosis, with the combination treatment further amplifying this effect, suggesting a cumulative apoptotic response over time (Figure 3A). Consistent with these findings, the apoptosis marker cleaved PARP was undetectable by western blot at 24 h in all conditions, while at 72 and 120 h, PARP cleavage was induced by the combination and, to a lower extent and depending on the cell line, by olaparib and/or Omomyc (Figure 3B). These results indicate that the pro-apoptotic effects of Omomyc and olaparib emerge progressively, with most apoptosis occurring from 72 h onwards, particularly under combination treatment. This also suggests that γH2AX accumulation after 24 h of treatment is mainly driven by DNA damage, with a minimal contribution from apoptosis (especially in the BRCA-mutated MX-1 and SUM149PT cells), and is exacerbated by the combination with olaparib in these TNBC PARPi-resistant models.

### Omomyc and olaparib cooperate to induce a durable anti-tumor response in TNBC CDXs

**Figure 4:**
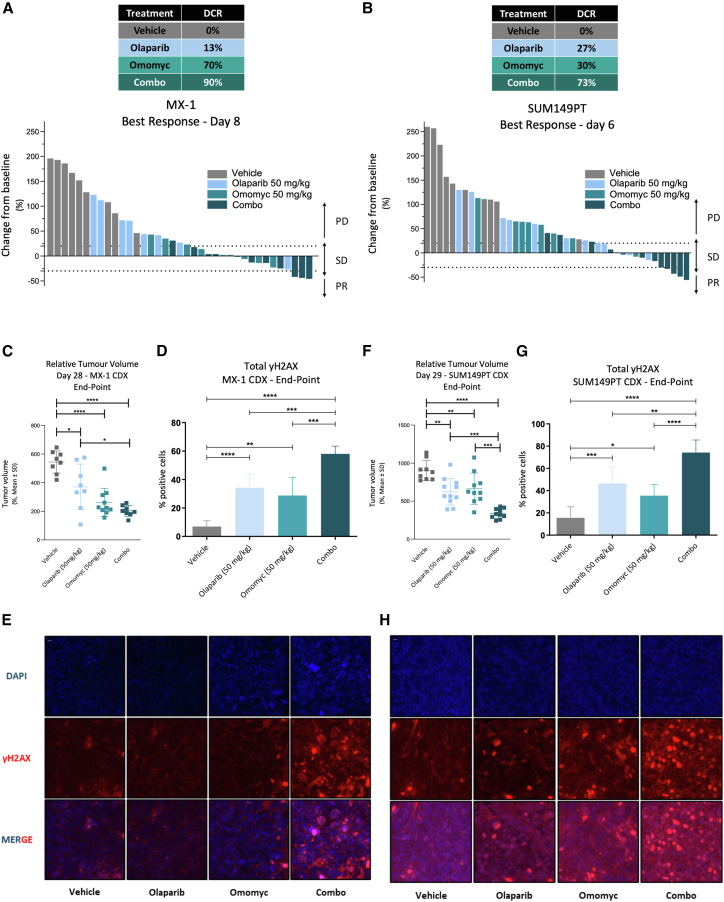
Omomyc cooperates with olaparib to induce durable anti-tumor response and DNA damage (A) Waterfall plot representing change from baseline (%, *y* axis) for each mouse on treatment after 8 days in the MX-1 CDX model. Treatments and their corresponding disease control rate (DCR; sum of stable disease [SD], partial response [PR], and complete response [CR]) are listed in a color-coded table. (B) Waterfall plot representing change from baseline (%, *y* axis) for each mouse on treatment after 6 days in the SUM149PT CDX model. Treatments and their corresponding DCR are listed in a color-coded table. (C) Dot plot representing relative tumor volume (*y* axis) at endpoint (28 days after treatment) in MX-1 CDX model. Four groups are represented: vehicle-treated mice, 50 mg/kg Omomyc, 50 mg/kg olaparib, or their combination. Error bars represent mean ± standard deviation. Asterisks represent statistical significance using an ordinary one-way ANOVA test with Tukey’s multiple comparisons test performed with GraphPad PRISM 9. ^∗^*p* < 0.05, ^∗∗^*p* < 0.01, and ^∗∗∗^*p* < 0.001; error bars, mean ± SD. (D) Bar chart representing the percentage of cells positive for γH2AX (*y* axis) in each treatment group (*x* axis) in MX-1 CDX model. Asterisks represent statistical significance using an ordinary one-way ANOVA test with Tukey’s multiple comparisons test performed with GraphPad PRISM 9. ^∗^*p* < 0.05, ^∗∗^*p* < 0.01, and ^∗∗∗^*p* < 0.001; error bars, mean ± SD. (E) Representative fluorescent microscopy images of formalin-fixed paraffin-embedded (FFPE) sections show the differences in γH2AX observed at endpoint in MX-1 CDX mice. Scale, 10 μm. (F) Dot plot representing relative tumor volume (*y* axis) at endpoint (28 days after treatment) in SUM149PT CDX model. Four groups are represented: vehicle-treated mice, 50 mg/kg Omomyc, 50 mg/kg olaparib, or their combination. Error bars represent mean ± standard deviation. Asterisks represent statistical significance using an ordinary one-way ANOVA test with Tukey’s multiple comparisons test performed with GraphPad PRISM 9. ^∗^*p* < 0.05, ^∗∗^*p* < 0.01, and ^∗∗∗^*p* < 0.001; error bars, mean ± SD. (G) Bar chart representing the percentage of cells positive for γH2AX (*y* axis) in each treatment group (*x* axis) in SUM149PT CDX model. Asterisks represent statistical significance using an ordinary one-way ANOVA test with Tukey’s multiple comparisons test performed with GraphPad PRISM 9. ^∗^*p* < 0.05, ^∗∗^*p* < 0.01, and ^∗∗∗^*p* < 0.001; error bars, mean ± SD. (H) Representative fluorescent microscopy images of FFPE sections show the differences in γH2AX observed at endpoint in SUM149PT CDX mice. Scale, 10 μm.

To translate these findings into an *in vivo* context, we generated orthotopic CDXs of MX-1 and SUM149PT cells, the two models that are BRCA mutated and would be candidates for PARPis in the clinic. Despite the expected PARPi resistance of these two cell lines, combination treatment of Omomyc and olaparib showed a dramatic therapeutic effect just after a week of treatment, where mice presented a synergistic increase in DCR as compared to each monotherapy in both models (Figures 4A and 4B). This anti-tumor response was durable and maintained throughout the 4 weeks of treatment. Indeed, analysis at the experimental endpoint showed that tumors in mice treated with either olaparib alone or Omomyc alone were much smaller than the tumors in untreated mice. When both drugs were used together, the tumors were even smaller (Figures 4C and 4F).

Moreover, fluorescent microscopy of paraffin-embedded tumors confirmed that, *in vivo*, Omomyc induces γH2AX foci appearance and that the combination with PARPis increases it further as compared to either monotherapy alone or vehicle-treated mice (Figures 4D, 4E, 4G, and 4H). This increase was observed in cells in all phases of the cell cycle but also in geminin+ cells, which specifically mark replicating cells in phase G2/S (Figures S4A–S4D).

### A stronger MYC transcriptional signature correlates with PARPi resistance in a large cohort of PDXs, where PARPis and Omomyc together elicit strong anticancer activity

**Figure 5:**
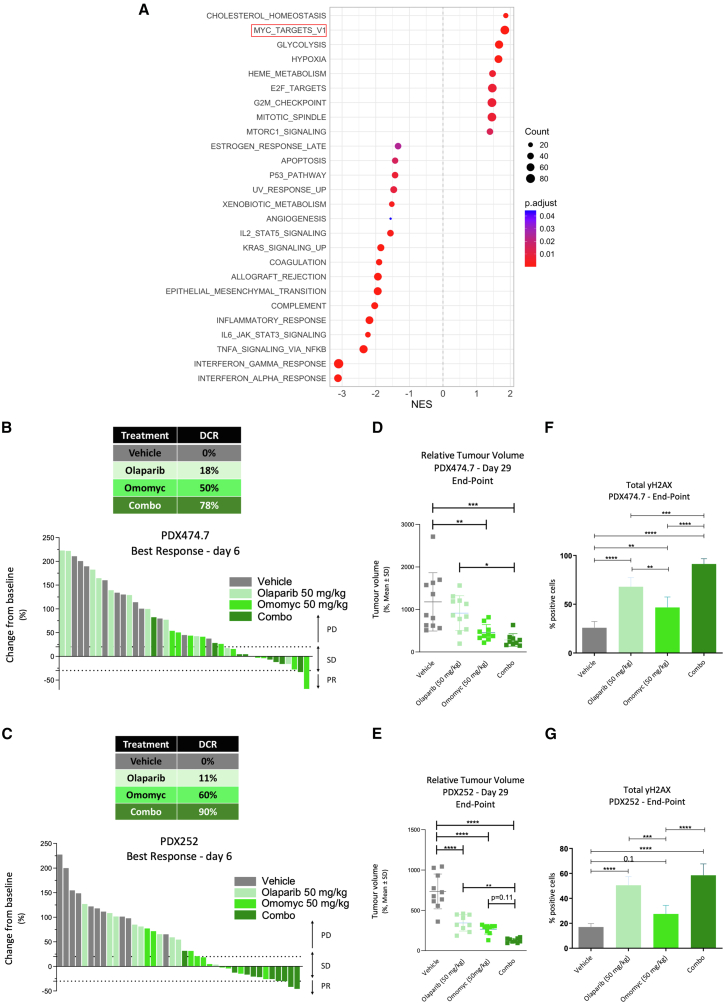
Stronger MYC transcriptional signature correlates with resistance to olaparib, which is reverted by Omomyc treatment (A) GSEA for the Hallmark gene sets regulated in olaparib-resistant PDXs versus olaparib-sensitive PDXs after treatment. The normalized enrichment scores (NESs) for the significantly enriched gene sets (FDR < 0.05) are presented in the bar plot. Color intensity represents the FDR, as indicated in the legend. Hallmark gene sets (MSigDB) were used for GSEA. FDR, false discovery rate. (B) Waterfall plot representing change from baseline (%, *y* axis) for each mouse on treatment after 6 days in the PDX474.7 model. Treatments and their corresponding disease control rate (DCR) are listed in the color-coded table. (C) Waterfall plot representing change from baseline (%, *y* axis) for each mouse on treatment after 6 days in the PDX252 model. Treatments and their corresponding DCR are listed in the color-coded table. (D) Dot plot representing relative tumor volume (*y* axis) at endpoint (28 days after treatment) in the PDX474.7 model. Four groups are represented: vehicle-treated mice, 50 mg/kg Omomyc, 50 mg/kg olaparib, or their combination. Error bars represent mean ± standard deviation. Asterisks represent statistical significance using an ordinary one-way ANOVA test with Tukey’s multiple comparisons test performed with GraphPad PRISM 9. ^∗^*p* < 0.05, ^∗∗^*p* < 0.01, and ^∗∗∗^*p* < 0.001; error bars, mean ± SD. (E) Dot plot representing relative tumor volume (*y* axis) at endpoint (28 days after treatment) in the PDX252 model. Four groups are represented: vehicle-treated mice, 50 mg/kg Omomyc, 50 mg/kg olaparib, or their combination. Error bars represent mean ± standard deviation. Asterisks represent statistical significance using an ordinary one-way ANOVA test with Tukey’s multiple comparisons test performed with GraphPad PRISM 9. ^∗^*p* < 0.05, ^∗∗^*p* < 0.01, and ^∗∗∗^*p* < 0.001; error bars, mean ± SD. (F) Bar chart representing the percentage of cells positive for γH2AX (*y* axis) in each treatment group (*x* axis) in the PDX474.7 model. Asterisks represent statistical significance using an ordinary one-way ANOVA test with Tukey’s multiple comparisons test performed with GraphPad PRISM 9. ^∗^*p* < 0.05, ^∗∗^*p* < 0.01, and ^∗∗∗^*p* < 0.001; error bars, mean ± SD. (G) Bar chart representing the percentage of cells positive for γH2AX (*y* axis) in each treatment group (*x* axis) in the PDX252 model. Asterisks represent statistical significance using an ordinary one-way ANOVA test with Tukey’s multiple comparisons test performed with GraphPad PRISM 9. ^∗^*p* < 0.05, ^∗∗^*p* < 0.01, and ^∗∗∗^*p* < 0.001; error bars, mean ± SD.

We analyzed a panel of 35 HR-altered PDXs and classified them according to their response to olaparib therapy (Figure S5A, adapted from Pedretti et al.^30^). Interestingly, bulk RNA sequencing revealed that the olaparib-resistant models (defined by progressive disease [PD] after 3 weeks of treatment) showed increased MYC transcriptional activity as compared to the sensitive models (defined by DCRs at the same time point) post-treatment (Figure 5A). Therefore, we selected two models within the resistant cohort (Figure S5B) with high MYC transcriptional signatures (Figure S5C) and tested their response to Omomyc, olaparib, and their combination. PDX474.7, one of the selected models, was the most resistant of the whole cohort and displayed a BRCA2 mutation, whereas the other one, PDX252, was a BRCA1 mutant, where olaparib slows down tumor growth without achieving disease control. As observed with CDXs, the combination of Omomyc and olaparib significantly increased the DCR as compared to the monotherapies or vehicle-treated groups in both models (Figures 5B and 5C). In more detail, olaparib did not have a significant impact on tumor growth in PDX474.7 and slowed down tumor growth in PDX252. However, the addition of Omomyc to olaparib translated into a significant therapeutic impact in both cases, significantly reducing their tumor volume at the experimental endpoint, after 4 weeks of treatment (Figures 5D and 5E).

Once again, the highest number of γH2AX foci was observed with the combination of both drugs as compared to each single agent alone or vehicle-treated tumors (Figures 5F and 5G). This increase was observed in cells in all phases of the cell cycle, including geminin+ cells (Figures S5D and S5E).

### Increased MYC transcriptional signature predicts poor response to PARPis in patients with metastatic breast cancer

**Figure 6:**
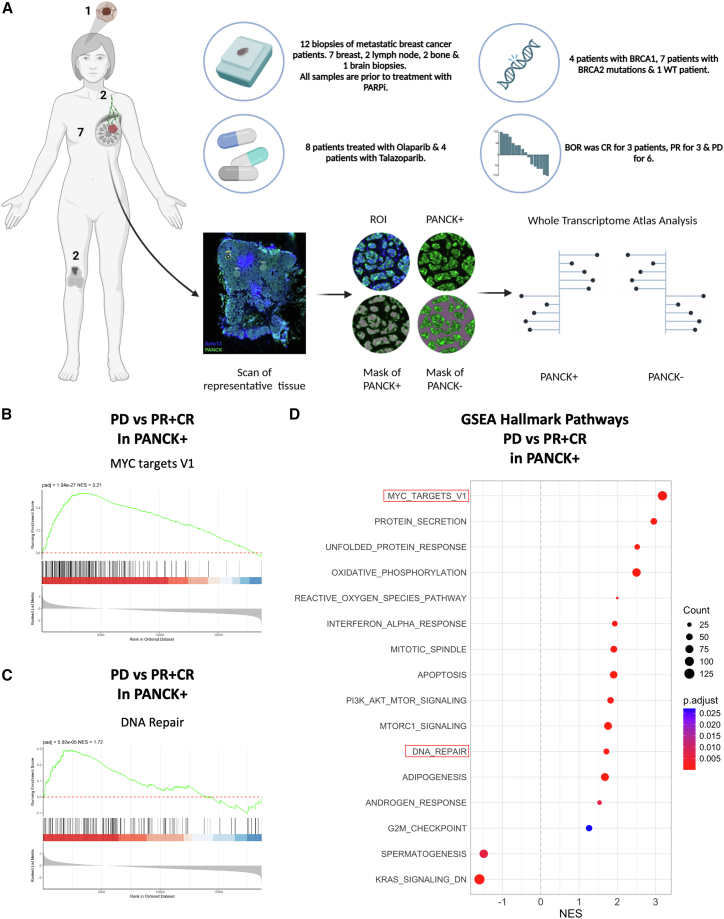
MYC transcriptional signature can serve as a predictor of PARPi response (A) Schematic representing DSP workflow. Created with BioRender.com. (B) GSEA comparing MYC target V1 gene set in PD as compared to PR+CR in PANCK+ regions. (C) GSEA comparing DNA repair gene set in PD as compared to PR+CR in PANCK+ regions. (D) GSEA for the Hallmark gene sets regulated in PD patients versus PR+CR patients in PANCK+ regions. The normalized enrichment scores (NESs) for the significantly enriched gene sets (FDR < 0.05) are presented in the bar plot. Color intensity represents the FDR, as indicated in the legend. Hallmark gene sets (MSigDB) were used for GSEA. FDR, false discovery rate.

In order to validate the correlation between MYC transcriptional signature and clinical response to PARPis, we analyzed 12 paraffin-embedded pre-PARPi treatment biopsies of patients with metastatic HR-altered breast cancer who were subsequently included in a clinical trial with PARPis (Figures S6A and S6B). Patients received either olaparib or talazoparib and were monitored for their best overall response (BOR) to therapy. Three patients had a complete response (CR), and three had a partial response (PR), while six displayed PD. Samples were collected from breast (primary tumor, seven), lymph nodes (metastasis, two), bone (metastasis, two), and brain (metastasis, one) (Figure 6A). DSP was used to selectively perform RNA sequencing on pan-cytokeratin (PANCK)+ tumor cells of each biopsy.

We first explored the fundamental relationship between MYC signaling and DNA repair, conducting correlation analyses across all patient samples. This analysis revealed a strong positive correlation between MYC target genes and DNA repair pathway genes in both PANCK+ and PANCK-negative (−) cells. When stratified by response groups, this positive correlation was maintained, confirming a fundamental biological relationship between MYC and DNA repair pathways in breast cancer (Figures S6C and S6E).

Subsequently, gene enrichment analysis (GSEA) was performed by grouping patients in objective responses (CR+PR) or PD. This comparison revealed that patients who progressed on PARPis had a significantly increased MYC transcriptional signature at baseline as compared to the patients who responded to the treatment (Figure 6B). This suggested that the MYC signature could potentially predict resistance to PARPi therapy. Patients who progressed on PARPis also presented with an increased DDR signature (Figure 6C), in line with the model for which MYC induces the DDR.

Of note, other pathways usually downregulated by Omomyc treatment and deeply connected with MYC biology, such as G2/M checkpoints and mitotic spindles,^31^ MTORC1/PI3K-AKT signaling,^32^^,^^33^ and protein secretion/unfolded responses,^34^ were upregulated in resistant patients as compared to responders (Figure 6D). Importantly, these changes were not observed in PANCK− regions (Figures S6F and S6G).

## Discussion

Our cells have evolved to make use of a complex infrastructure, the DDR, to preserve genomic integrity against the continuous threats that loom over our genetic material.^35^ The importance of the DDR is highlighted by the central role its defects play in human diseases,^36^ including malignant transformation.^37^ Among the oncological indications most renowned for DDR defects, there is hereditary TNBC, often caused by mutations in the HR-related gene *BRCA1* or *BRCA2*. These mutations render cells incapable of successfully repairing DSBs in DNA, driving genomic instability. This increases the cumulative chance of developing breast cancer by the age of 70 years from 40% to 87% for *BRCA1* and from 27% to 84% for *BRCA2* carriers.^38^

TNBC is characterized by the absence of the three main druggable receptors within breast cancer cells: estrogen receptor (ER), progesterone receptor (PR), and HER2. This feature makes TNBC extremely challenging to treat, as demonstrated by its poorer survival outcomes in advanced stages when compared to any other breast cancer molecular subtype.^39^ Today, there are few treatment options for TNBC beyond cytotoxic chemotherapy.^18^ The evolving treatment landscape for metastatic TNBC now encompasses immune checkpoint inhibitors in PD-L1+ patients^19^ and antibody-drug conjugates such as sacituzumab govitecan for pre-treated disease.^20^ Another possible alternative is PARPis, which emerged as a breakthrough therapeutic strategy in the early 2000s^22^ by exploiting the defects in *BRCA1* and *BRCA2*. Until then, mutations in those genes were considered a cancer prophecy waiting to happen, but PARPis transformed a bad omen into a therapeutic opportunity. Indeed, by inhibiting PARP1 and impairing a cell’s ability to repair single-strand DNA breaks (SSBs), PARPis took advantage of classical synthetic lethality in cancer treatment.

Unfortunately, just a few years after the approval of olaparib for BRCA-mutated breast cancer, the emergence of complex resistance mechanisms appears to hinder the full therapeutic potential of PARPis.^40^ Specific examples of such mechanisms are the restoration of HR repair capacity through secondary BRCA mutations, enhanced drug efflux via ATP-binding cassette transporters, stabilization of stalled replication forks through RAD51 recruitment, and reduced PARP trapping at sites of DNA damage, all of which collectively revert the synthetic lethal interaction underlying the therapeutic efficacy of PARPis.^41^

MYC, one of the most-wanted targets in oncology,^42^ is disproportionally elevated in TNBC as compared to other breast cancers^43^ and has a controversial role within genomic instability.^4^ Indeed, MYC overexpression has been linked by many to stalled replication forks and increased DNA damage,^5^ while others have shown that it promotes DNA repair.^6^

In this article, we show that Omomyc, the only direct MYC inhibitor to reach a phase 2 clinical trial so far, can induce DDR defects and DNA damage in cancer cells and propose it as a cutting-edge therapeutic opportunity to overcome PARPi resistance. In more detail, we show that MYC inhibition (MYCi) by Omomyc causes transcriptional repression of genes and gene sets related to DDR and HR, confirming the role of MYC in controlling DNA repair pathways in TNBC. In fact, our data indicate that, in the TNBC PARPi-resistant context, MYC protects against DNA damage, increasing the DDR and contributing to HR proficiency. On the contrary, Omomyc downregulates key proteins such as PARP1 and RAD51, which serve as fundamental gatekeepers of DNA repair: PARP1 plays a central role in SSB repair, facilitating base excision repair and preventing replication-associated collapse,^44^ while RAD51 is the essential last recombinase in HR, where it mediates the search for a homologous DNA template and promotes strand invasion to ensure error-free DSB repair.^45^ The fact that Omomyc downregulates these two major players within the DDR is consistent with prior evidence suggesting that MYC-driven tumors exhibit heightened dependence on these repair factors, making them particularly vulnerable to interventions targeting the DDR.^46^ In this context, Omomyc acts as a DNA-damaging agent, not by directly inducing lesions but, more likely, by impairing the cell’s ability to resolve them. This prompted us to combine this MYC inhibitor with PARPis.

Our findings demonstrate that the combination of MYCi by Omomyc and PARPis by olaparib or talazoparib is synergistic at multiple concentrations *in vitro*. When combining Omomyc and PARPis, we observed increased γH2AX, a marker of DNA DSBs and chromatin remodeling, already at 24 h post-treatment. However, γH2AX is not exclusively a marker of DNA damage and is often associated with apoptosis and particularly DNA fragmentation.^27^ To assess whether such an increase was indeed related to DNA damage and not apoptosis, we employed Annexin V/PI staining and cleaved PARP western blotting, which both confirmed negligible apoptosis at 24 h, strongly suggesting that, at this early time point, γH2AX mostly reflects DNA damage. Apoptosis instead becomes more pronounced at 120 h, indicating a progressive commitment to cell death.

In order to study the *in vivo* effect of MYCi in the context of PARPi resistance, we made use of CDXs and PDXs of BRCA-mutated PARPi-resistant models and recapitulated the anti-tumor efficacy observed *in vitro*. Mechanistically, the increase in γH2AX+ cells in the combination group suggests a direct relationship between increased DNA damage and therapeutic response.

Patient-derived samples also shed light on the fundamental role of oncogenic MYC within PARPi resistance. First, a large cohort composed of 35 HR-altered metastatic breast cancer PDXs treated with olaparib showed that olaparib-resistant models had a substantial increase in the MYC transcriptional signature as opposed to the ones that responded to treatment. Of note, we believe that MYC transcriptional activity is a better readout of MYC addiction than its absolute levels, as previously proposed.^16^

Finally, to see whether an oncogenic MYC transcriptional signature could have a predictive value in patients with TNBC treated with olaparib or talazoparib, we collected 12 pre-treatment biopsies of patients with metastatic breast cancer enrolled in a PARPi clinical trial and matched them retrospectively with the patients’ best objective response. We divided the patients between those who achieved an objective response (PR+CR) and those who progressed (PD) and performed a DSP analysis comparing these two treatment groups. According to our preclinical findings, patients who did not respond to PARPis had a significantly higher MYC signature in PANCK+ cancer cells than responders. Together with MYC transcriptional activity, we also present the DDR as a prognostic indicator of PARPi response by showing, through DSP, that the expression of the DNA repair pathway gene set positively correlated with MYC targets and was also enriched in non-responders as compared to responders. In this analysis, both BRCA1 and BRCA2 patients were included, and there was no difference in the predictive value of the MYC signature between the two mutations. Analysis of other upregulated cancer pathways also revealed a connection with MYC, reinforcing its role as a master regulator of cellular metabolism,^47^ as well as a promoter of mitochondrial biogenesis and energy production.^48^ Furthermore, the upregulation of protein secretion and unfolded protein response pathways aligns with MYC’s known function in driving protein synthesis and processing machinery,^34^ as cancer cells must cope with increased protein production demands. The observed activation of G2/M checkpoint and mitotic spindle pathways is also consistent with MYC’s fundamental role in cell cycle regulation.^31^ Notably, we detected enhanced MTORC1 and PI3K/AKT/mTOR signaling, highlighting the complex regulatory network where MYC acts both upstream and downstream of these pathways.^32^ This signaling cascade likely creates a feedforward loop that sustains the observed metabolic and proliferative phenotypes.

Importantly, these differences occurred only in PANCK+ cancerous regions of the biopsies and not in the PANCK− stromal areas, highlighting the importance of choosing spatial transcriptomics, instead of bulk strategies, to guarantee that cancer-specific differences are not diluted or missed. The convergence of these multiple MYC-driven pathways strongly supports the rationale for MYC inhibition by Omomyc as a therapeutic strategy in PARPi-resistant patients.

Of note, DSP analysis performed in patients participating in the Omomyc first-in-human study revealed that DNA repair was shut down in on-treatment versus pre-treatment biopsies, demonstrating that the effects of Omomyc on DNA repair that we describe herein in preclinical models are, at least in part, recapitulated in the clinic.^16^

For the sake of completeness, it should be mentioned that MYC has been suggested as an attractive therapeutic target in the context of DDR-related diseases. For instance, MYC has been shown to control expression of *ABCG2*,^40^ a drug-efflux transporter gene related to resistance to PARPis.^49^ In addition, PARPis do not completely block PARP activity, allowing compensatory mechanisms, such as poly (ADP-ribose) glycohydrolase (PARG) loss, to restore PARP1-dependent DNA damage signaling.^50^ Since MYC directly interacts with the *PARP1* promoter controlling its expression,^51^ such compensatory mechanisms could be prevented by MYCi. Moreover, a common mechanism of resistance to PARPis is the reactivation of *BRCA1*, *BRCA2*, or *RAD51* function,^52^^,^^53^^,^^54^ and MYC has been shown to regulate their expression.^6^^,^^55^ Lastly, MYC directly recruits and interacts with DNA *TOP2A* to form the specialized “topoisome” complex that facilitates DNA unwinding and manages topological stress at key genomic regions during transcription and replication. This TOP2A-MYC interaction supports high transcriptional demands in cancer cells, though excessive activity triggers DNA damage through DSBs.^56^

Overall, by using multiple TNBC preclinical and clinical models, in this study, we revealed the role of MYC as both a driver of PARPi resistance and a predictor of poor response to therapy and demonstrated how MYCi by Omomyc can induce DNA damage and offer the opportunity for combination with PARPis even in PARPi-resistant disease. This, added to the fact that Omomyc has been deemed safe and demonstrated target engagement in the clinical setting, prompts further clinical exploration of its combination with PARPis in TNBC and, possibly, other oncological indications. Indeed, future experimentation will be crucial to verify whether this combination strategy could be expanded to other BRCA-mutated tumors and beyond.

### Limitations of the study

This study has limitations that should be acknowledged. For instance, the sample size of our patient cohort was small (*n* = 12), and larger, independent studies will be necessary to validate MYC as a predictive biomarker for PARPi resistance and to confirm the clinical relevance of our findings.

A comprehensive genomic characterization of our PDX models, including analysis of MAPK pathway alterations, broader DNA damage repair genes, or potential BRCA reversion mutations, should be performed to shed light on the specific resistance mechanisms of the different models.

It should also be noted that the immune microenvironment contexture in our patient samples could contribute to their sensitivity to treatments. Such analysis would require larger cohorts and targeted immune profiling approaches to comprehensively characterize immune cell populations and their functional states, which is an important area for future investigation.

Finally, in this study, clinical validation was restricted to pre-treatment samples from a single cohort. Analysis of independent patient populations and matched pre- and post-treatment samples would strengthen the clinical utility of our biomarker findings.

## Resource availability

### Lead contact

Further information and requests for resources and reagents should be directed to and will be fulfilled by the lead contact, Laura Soucek (lsoucek@vhio.net).

### Materials availability

All materials and resources presented in this manuscript are being made available.

### Data and code availability

• Microarray data from Massó-Vallés et al.^11^ were obtained from Gene Expression Omnibus (GEO) (RRID: SCR_005012): GSE174447 at https://www.ncbi.nlm.nih.gov/geo/query/acc.cgi?acc=GSE174447.
• RNA sequencing *in vitro* data generated in this study are deposited in GEO: GSE309250 at https://www.ncbi.nlm.nih.gov/geo/query/acc.cgi?acc=GSE309250.
• RNA sequencing PDX data generated in Pedretti et al.^30^ were obtained from Zenodo at https://zenodo.org/records/14654577.
• DSP data generated in this study are deposited in EGA (EGAS50000001347) at https://ega-archive.org/studies/EGAS50000001347.
• Scripts and code were written for R and are available upon request.

## Acknowledgments

We acknowledge the kind support from the Vall d’Hebron Institute of Oncology and the Cellex Foundation for providing research facilities and equipment. We thank the Spanish Association of Metastatic Breast Cancer for the Chiara Giorgetti Award 2022, 10.13039/501100004587Instituto de Salud Carlos III (FI20/00274 – F.G., PFIS doctoral bursary); Ministerio de Ciencia e Innovación (Retos de Colaboración RTC2019-007067-1 and Líneas Estratégicas PLEC2021-007959); Generalitat de Catalunya (AGAUR 2017/SGR 537 and 2021/SGR 01509); EDIReX (H2020 INFRAIA 731105-2); the European Research Council (ERC-2023-ADG 101142260); the Spanish Ministry of Science and Innovation and the European Union through the NextGenerationEU program, in the context of the Plan de Recuperacion, Transformacion y Resiliencia (RETOS; CPP2022-009808); and the Fero Foundation for funding support.

## Author contributions

F.G., D.M.-V., and L.S. contributed to the conceptualization; F.G., I.G.-L., A.H.-R., S.C.-S., M.F.Z.-F., M.A., F.P., S.M.-M., H.T., V.C.C., L.N., D.M.-V., and L.S. contributed to the methodology; F.G., I.G.-L., A.H.-R., M.F.Z.-F., M.A., F.P., J.G., L.F., E.S., S.L.-E., O.R., M.G., and D.M.-V. contributed to the investigation; A.R.-H., F.B.-M., A.L.-G., J.B., A.P., and V.S. contributed to the clinical sample acquisition and data curation; L.S. contributed to funding acquisition and project administration; L.S. and D.M.-V. contributed to supervision; and F.G., I.G.-L., S.C.-S., S.M.-M., M.-E.B., J.R.W., D.M.-V., and L.S. contributed to the writing.

## Declaration of interests

F.G., D.M.-V., and L.S. are inventors of a European Patent (EP23382554), licensed to Peptomyc S.L., related to this topic. S.C.-S., S.M.-M., M.A., H.T., J.G., L.F., S.L.-E., V.C.C., M.-E.B., D.M.-V., and L.S. are or were employees of Peptomyc S.L. M.F.Z.-F., S.C.-S., L.F., and J.R.W. are shareholders of Peptomyc S.L. L.S. and M.-E.B. are cofounders and shareholders of Peptomyc S.L. V.S. reports non-commercial research funding with AstraZeneca, Circle Pharma, Astex, and DebioPharm and has served as an advisor for GSK. A.L.-G. and V.S. are current employees of AstraZeneca.

## STAR★Methods

### Key resources table

**Table:** 

REAGENT or RESOURCE	SOURCE	IDENTIFIER
**Antibodies**		

Rabbit monoclonal anti-GAPDH	Cell Signaling Technology	Cat# 2118; RRID:AB_561053
Rabbit monoclonal anti-RAD51	Abcam	Cat# ab133534; RRID:AB_2722613
Rabbit polyclonal anti-PARP1	Cell Signaling Technology	Cat# 9542; RRID:AB_2160739
Rabbit polyclonal anti-Cleaved PARP	Cell Signaling Technology	Cat# 9545; RRID:AB_2283565
ECL anti-rabbit IgG-HRP secondary antibody	GE Healthcare	Cat# NA934; RRID:AB_772206
Anti-phospho-histone H2A.X (Ser139)-FITC	Merck/Millipore	Cat# 16-202A
Mouse IgG-FITC isotype control	Merck/Millipore	Cat# 12-487
Anti-γH2AX (Mouse monoclonal, clone JBW301)	Millipore	Cat# 05-636-I; RRID:AB_309864
Anti-geminin (Rabbit polyclonal)	ProteinTech Group	Cat# 10802-1-AP; RRID: AB_2110945
Anti-geminin (Mouse monoclonal)	NovoCastra NCL-L	Cat# 6096384
Anti-BRCA1 (Mouse monoclonal, D-9)	Santa Cruz	Cat# sc-6954; RRID:AB_626761
Alexa Fluor 488 anti-mouse secondary antibody	Invitrogen	Cat# A-11001; RRID:AB_2534069
Alexa Fluor 568 anti-rabbit secondary antibody	Invitrogen	Cat# A-11011; RRID:AB_143157
Annexin V-APC	Biolegend	Cat# 640920

**Biological samples**		

FFPE HR-altered metastatic breast cancer biopsies	Vall d'Hebron University Hospital	N/A
Patient-derived xenograft tumor biopsies	Vall d'Hebron University Hospital	N/A

**Chemicals, peptides, and recombinant proteins**		

Omomyc	Peptomyc S.L.	Proprietary
Olaparib	Medchemexpress	Cat#HY-1 0162
Talazoparib	Medchemexpress	Cat# HY-16106

**Critical commercial assays**		

MycoAlert	Lonza	Cat# LT07-118
DC Protein Assay	Bio-Rad	Cat# 5000112
eBioscience™ Transcription Factor Fixation/Permeabilization kit	Thermo Fisher Scientific	Cat# 00-5523-00
RNeasy Mini Kit	Qiagen	Cat# 74106
AlamarBlue	Thermo Fisher Scientific	Cat# DAL1025

**Deposited data**		

Microarray data	Massó-Vallés et al.^11^	Gene Expression Omnibus (GEO) (RRID:SCR_005012) at GSE174447; https://www.ncbi.nlm.nih.gov/geo/query/acc.cgi?acc=GSE174447
RNA sequencing data – *in vitro*	This paper	GEO: GSE309250; https://www.ncbi.nlm.nih.gov/geo/query/acc.cgi?acc=GSE309250
RNA sequencing data - PDXs	Pedretti et al.^30^	Zenodo: https://zenodo.org/records/14654577
Digital spatial profiling data	This paper	EGA: EGAS50000001347; https://ega-archive.org/studies/EGAS50000001347

**Experimental models: Cell lines**		

MDA-MB-231	ATCC	Cat# HTB-26; RRID:CVCL_0062
MX-1	CLS Cell Lines Service (Cytion)	Cat# 300296; RRID:CVCL_4774
SUM149PT	BIOIVT, Asternand	Cat# HUMANSUM-0003004; RRID:CVCL_3422
SUM1315MO2	BIOIVT, Asternand	Cat# HUMANSUM-0003002; RRID:CVCL_5589

**Experimental models: Organisms/strains**		

BALB/c nude female mice	Janvier	RRID:IMSR_JCL:JCL:mID-0001
NMRI nude female mice	Janvier	RRID:IMSR_TAC:nmrinu

**Software and algorithms**		

GSEA	Broad Institute	RRID:SCR_003199; v3.0
GraphPad Prism 9	GraphPad Software	RRID:SCR_002798; v9.0
R	R Foundation	RRID:SCR_001905; v4.1.3, v4.4.1
RStudio	Posit	RRID:SCR_000432; v2024.12.0 + 467
FCS Express	*De Novo* Software	RRID:SCR_016431
CytExpert	Beckman Coulter	RRID:SCR_017217
SynergyFinder.org	Web tool	RRID: SCR_026127
MSigDB	Broad Institute	RRID:SCR_016863; 2022 version
BioRender	Web tool	RRID:SCR_018361

**Other**		

Millicell EZ 8-well slides	Merck Millipore	Cat# PEZGS0816
iBlot3 Dry Blotting System	Life Technologies	Cat# IB23001
Vi-CELL	Beckman Coulter	RRID:SCR_019664
BD FACSCanto	BD Biosciences	RRID:SCR_018065
CytoFLEX LX	Beckman Coulter	RRID:SCR_025067
TECAN Spark microplate reader	Tecan	RRID:SCR_021897
iBRIGHT CL 1500	Invitrogen	RRID:SCR_026565
Nikon Eclipse TI inverted epifluorescence microscope	Nikon	RRID:SCR_021242

### Experimental model and study participant details

#### Cell lines

MDA-MB-231 cells were purchased from ATCC (catalog no. HTB-26), MX-1 cells were purchased from CLS Cell Lines Service (now Cytion, Eppelheim, Germany, catalog no. 300296), SUM149PT (catalog no. HUMANSUM-0003004, BIOIVT, Asternand) and SUM1315MO2 (catalog no. HUMANSUM-0003002, BIOIVT, Asternand) cell lines were kindly provided by Dr Violeta Serra. MDA-MB-231 and MX-1 cells were maintained in DMEM/F12 (Life Technologies) with 10% FBS, while SUM149PT and SUM1315MO2 were cultured in F12 (Life Technologies) supplemented either with 5μg/ml insulin, 10mM HEPES, 1μg/ml hydrocortisone and 1% penicillin/streptomycin for the first, and 5μg/ml insulin, 10mM HEPES, 10ng/ml EGF and 1% penicillin/streptomycin for the latter. Cell lines were authenticated at the Vall d'Hebron Institute of Research (VHIR) High Technology Unit (UAT) by microsatellite analysis. Mycoplasma testing (MycoAlert, Lonza) was performed periodically. All experiments were performed within 10 passages after thawing.

#### Animal studies

All animal studies were conducted in accordance with the ARRIVE guidelines and the 3Rs principles (Replacement, Reduction, and Refinement). Mice were housed and treated following protocols approved by the Ethical Committee for the Use of Experimental Animals (CEEA, protocol 12/22) at VHIO-VHIR, Barcelona, Spain and by the Commission of Animal Experimentation (code 11818) at Generalitat de Catalunya, Departament d’Acció Climàtica, Alimentació i Agenda Rural. All experiments were performed using female mice to reflect the predominant patient population in breast cancer.

Cell line-derived xenografts (CDXs) were generated by orthotopically implanting MX-1 or SUM149PT cells into BALB/c nude female mice (Janvier RRID:IMSR_JCL:JCL:mID-0001). A suspension of 1.5 × 10^6^ to 5 × 10^6^ tumor cells, depending on the model, mixed 1:1 with Matrigel, was injected between the fourth and fifth right mammary fat pads after a small incision. The wound was sutured with nonabsorbable thread (6–0), and before surgery, mice were anesthetized with 2.5% isoflurane.

For patient-derived xenografts (PDXs), tumor biopsies from metastatic breast cancer patients at Vall d’Hebron University Hospital were expanded in NMRI nude female mice (Janvier RRID:IMSR_TAC:nmrinu). Tumors were excised, divided, and implanted subcutaneously into new recipient mice. Mice were anesthetized with 2.5% isoflurane, and a small dorsolateral flank incision was made to insert the tumor fragment before sealing with Histoacryl.

Tumor growth was monitored twice per week by caliper measurements, and tumor volume was calculated as (length × width^2^)/2. Once tumors reached 100 mm^3^, mice were randomised into four treatment groups (9–12 animals per group): vehicle, Omomyc (50 mg/kg), Olaparib (50 mg/kg), and the combination therapy. Treatments were administered for 4 weeks (Omomyc, intravenous once a week, while Olaparib by oral gavage 6 times a week), after which mice were euthanised by cervical dislocation just after an exsanguination by cardiac puncture. Tumors and other organs that are common metastatic sites in breast cancer (lymph nodes, lungs, liver, skin and brain) were excised, weighed, fixed in 4% buffered formaldehyde for 24 h, transferred to 70% ethanol for an additional 24 h, and embedded in paraffin. Animals were excluded from the study if their tumor volumes significantly deviated from the cohort average, as determined by statistical outlier analysis using GraphPad Prism 9.0.

#### Human samples

Formalin-fixed paraffin-embedded (FFPE) tumor biopsies were obtained from 12 female patients with HR-altered metastatic breast cancer prior to PARP inhibitor treatment. All patients were subsequently enrolled in clinical trials receiving either Olaparib or Talazoparib as monotherapy. Patient samples were collected from the following anatomical sites: breast (primary tumor, *n* = 7), lymph nodes (metastasis, *n* = 2), bone (metastasis, *n* = 2), and brain (metastasis, *n* = 1).

Patients were stratified based on best overall response (BOR) to PARPi therapy: three patients achieved complete response (CR), three achieved partial response (PR), and six displayed progressive disease (PD). For differential gene expression analysis, patients were grouped into objective responders (CR + PR, *n* = 6) versus progressive disease (PD, *n* = 6).

All samples were collected with informed consent, and the study was conducted in compliance with the Vall d'Hebron University Hospital Clinical Research Ethics Committee (CEIm) approval. As breast cancer predominantly affects females and reflects the patient population enrolled in the associated clinical trials, only female patient samples were included in this study.

### Method details

#### Microarray analysis and RNA sequencing

For microarray analysis, MDA-MB-231 cells were seeded into 10 6cm dishes. The next day, 5 plates were treated with 20μM Omomyc and the remaining 5 with an equivalent volume of vehicle. After 72 h, plates were washed twice with PBS and frozen at −80°C until processing. RNA was extracted with TRIzol reagent (Invitrogen) according to the manufacturer’s instructions. The quality of RNA was confirmed with Agilent 2100 Bioanalyzer. Clariom S Human HT microarray plate (Applied Biosystems) was processed at Vall d’Hebron Institute of Research (VHIR)’s High Technology Unit. The microarray data were analyzed with Partek Genomics Suite software, v7.18. Gene set enrichment analysis (GSEA) was performed using publicly available software provided by the Broad Institute (RRID:SCR_003199, version 3.0) with the Hallmarks, Curated, Motif, GO, Oncogenic Signatures, and Immunological Signatures gene sets from the Molecular Signature Database (MSigDB; www.broad.mit.edu/gsea). The number of permutations was set to 1,000, and the genes were ranked according to Signal2Noise.

For *in vitro* RNA sequencing, MDA-MB-231 cells were treated with 30μM Omomyc for 120 h. RNA extraction and sequencing have been performed by qGenomics (Barcelona, Spain). Analysis was performed in R (v4.1.3) using voom+limma (v3.50.1). Genes with <15 counts across ≥6 samples were filtered out. Data were normalized using trimmed mean of M-values (TMM) in edgeR for unsupervised analyses. For differential expression, log2-counts-per-million were modeled using voom precision weights, with RINDef as a covariate. Genes with FDR-adjusted *p*-value <0.05 were considered differentially expressed. Pathway enrichment was conducted using pre-ranked GSEA (clusterProfiler v4.2.2), utilizing MSigDB (2022) gene sets. Ranks were generated by multiplying the -log(*p*-value) by the sign of the logFC for each gene. The expression heatmaps were generated using R version 4.4.1 in R Studio version 2024.12.0 + 467, with the pheatmap package version 1.0.12.

For *in vivo* bulk RNA sequencing Total RNA was extracted from fresh-frozen tumors using RNeasy Mini Kit (Qiagen) following manufacturer’s protocol, with tissue homogenization performed using POLYTRON PT system (Kinematica). RNA quantity and purity were assessed via NanoDrop ND-1000 spectrophotometer (Thermo Scientific). RNA-seq was performed at CNAG-CRG (Barcelona, Spain). Samples with RIN>7 (RNA 6000 Nano Bioanalyzer 2100 Assay, Agilent) were processed using KAPA Stranded mRNA-Seq Kit (Roche) and sequenced on Illumina NovaSeq 6000 (2x40bp paired-end). Reads were mapped to combined human (GRCh37) and mouse (GRCm38) reference genomes using STAR (v2.5.7a) with ENCODE parameters. Gene quantification used combined human GENCODE v39 and mouse GENCODE M27 annotations via RSEM (v1.3.0). Only human-aligned reads were analyzed further. Differential expression analysis was performed on protein-coding genes after noise removal (noisyr v1.0.0). DESeq2 (v1.36.0) was used with the model: Y ∼ Group + Patient, where Group combined treatment (vehicle/Olaparib) and PARPi-response (sensitive/resistant). Genes with BH FDR <0.05 and |log2FC| > 0.575 were considered differentially expressed.

#### Western blots

Cells were cultured in 6-well plates and treated with either vehicle, 30μM of Omomyc, 5μM of Olaparib or combination of both drugs for 24, 72 or 120 h. Then, cells were trypsinised, centrifuged, washed twice with PBS and kept at −80°C. Subsequently, cell pellets were lysed with RIPA buffer supplemented with protease and phosphatase inhibitors (Life Technologies) and the protein fraction collected. Proteins were quantified by the DC Protein Assay (Bio-Rad) and absorbance at 560 nm was measured with a spectrophotometer (Victor3, PerkinElmer or TECAN Spark, Life Sciences). 10 to 30 μg of protein extract in Laemmli buffer + DTT were run on 10% or 12% precast gels (Life Technologies). Proteins were transferred to PVDF membranes by the iBlot3 Dry Blotting System (Life Technologies) and membranes were stained with Ponceau red to verify proper transfer. After washing with PBS +0.1% Tween and blocking with 5% milk, membranes were incubated overnight at 4°C with the following primary antibodies: rabbit monoclonal anti-GAPDH (Cell Signaling Technology, catalog no. 2118, 1:50,000); rabbit monoclonal anti-RAD51 (Abcam, catalog no. ab133534, 1:1,000); rabbit polyclonal anti-PARP1 (Cell Signaling Technology, catalog no. 9542, 1:1,000); rabbit polyclonal anti-Cleaved PARP (Cell Signaling Technology, catalog no. 9545, 1:1,000). Membranes were washed with PBS-Tween and incubated for 1 h at room temperature with the ECL anti-rabbit IgGHRP secondary antibody (GE Healthcare, catalog no. NA934, 1:5,000). Membranes were washed twice with PBS-Tween and once with PBS and then incubated for 5 min with SuperSignal West Pico Chemiluminescent Substrate (Thermo Fisher Scientific) before revealing with IBRIGHT CL 1500 (Invitrogen).

#### Flow cytometry

Cells were seeded in 6-well plates and treated with vehicle and 30μM of Omomyc alone, or with 5μM of Olaparib and their combination. For DNA damage assessment, cells were harvested at 24- and 72-h post-treatment. For apoptosis analysis, additional timepoints at 120-h were included. Following treatment, cells were collected, including media and PBS wash, trypsinised (0.25% trypsin, 30 min), and centrifuged. Cell counts were performed using Vi-CELL (Beckman Coulter).

For γH2AX detection, 1×10^5^ cells were stained with BD Horizon Fixable Viability Stain 510 (15 min), washed with PBS-Azide (PBS containing 0.1% sodium azide, 1% BSA), and stained using the eBioscience Transcription Factor Fixation/Permeabilization kit according to manufacturer’s instructions. Briefly, cells were blocked (1% BSA, 5% horse serum, 0.5% 0.5M EDTA) for 10 min at room temperature. Then, cells were fixed and permeabilized followed by incubation with anti-phospho-histone H2A.X (Ser139)-FITC or mouse IgG-FITC isotype control (0.0006mg/ml per sample, Merck/Millipore) for 30 min. Acquisition was performed on a BD FACSCanto (BD Biosciences) flow cytometer and the FCS Express software was used for analysis.

For cell cycle analysis, cells were fixed with ice-cold ethanol 100% and propidium iodide (1μL/sample, 100mg/L) was used to stain the cells for 15 min. Acquisition was performed on a BD FACSCanto flow cytometer and the FCS Express software was used for analysis.

For apoptosis detection, 1×10^5^ cells were stained with Annexin V-APC (1.5μL/sample, Biolegend) and propidium iodide (1μL/sample, 100mg/L) for 15 min in Annexin binding buffer (Invitrogen). Acquisition was conducted using a CytoFLEX LX flow cytometer (Beckman Coulter) and CytExpert software was used for analysis.

#### Drug sensitivity and synergy analysis

Cells were seeded in 96-well plates (2,500-13,000 cells/well, based on cell line characteristics) and treated the following day. For single-agent studies, cells were exposed to decreasing concentrations of Olaparib (100 μM–0.39 μM) or Talazoparib (100 nM–0.39 nM) in 1:2 serial dilutions. After 120 h, AlamarBlue (Thermo Fisher Scientific) was added to the wells, and fluorescence (590 nm) was measured after 2 h using a Spark microplate reader (Tecan). GI 50 values were calculated using GraphPad Prism 9 (RRID:SCR_002798).

For combination studies, cells were treated with increasing concentrations of Olaparib plus Omomyc or Talazoparib plus Omomyc. Drug interaction was evaluated using the HSA (Highest Single Agent) scoring model on SynergyFinder.org.^57^ HSA scores above 10 indicate synergistic interactions, between −10 and 10 suggest additive effects, and below −10 indicate antagonism.

#### Immunofluorescence

Cells were cultured in Millicell EZ 8-well slides (Merck Millipore) (5,000–20,000 cells/well, based on cell line characteristics). At 24 h after treatment with vehicle, 30μM of Omomyc, 5μM of Olaparib or their combination, cells were washed with PBS, fixed with 4% paraformaldehyde for 15 min, and permeabilized with 0.5% Triton X-100 in PBS for 5 min. Samples were blocked for 40 min with blocking buffer (2% BSA, 0.2% Triton X-100, 1% goat serum in PBS). Fixed cell samples were stored at 4°C until further processing.

For tissue analysis, formalin-fixed paraffin-embedded (FFPE) tumor tissues were collected at endpoint, fixed in 4% formalin for 24 h, and further preserved in 70% ethanol for an additional 24 h. FFPE blocks were generated by the Vall d’Hebron Institute of Oncology (VHIO) Molecular Oncology Group. Tissue sections (3 μm) were deparaffinised and rehydrated through xylene and graded ethanol series. Antigen retrieval was performed using DAKO Antigen Retrieval Buffer pH 9 in a microwave (20 min heating, 30 min cooling). Sections were permeabilised and blocked with DAKO Wash Buffer containing 1% BSA.

Primary antibodies against γH2AX (Mouse monoclonal, clone JBW301, Millipore #05-636-I, 1:200) were used for both cells and tissue sections, whereas anti-geminin (Rabbit polyclonal, ProteinTech Group, 10802-1-AP, 1:400) was used exclusively in tumor tissue sections. Antibodies were applied for 1 h at room temperature. After washing, cells were incubated with Alexa Fluor 488 anti-mouse secondary antibody, whereas tissue sections received both Alexa Fluor 488 anti-mouse and Alexa Fluor 568 anti-rabbit antibodies (all 1:500) for 30–40 min. All samples were mounted using ProLong Gold antifade reagent with DAPI (Invitrogen). Cells were stored at 4°C, while FFPE tissue sections were stored at −20°C until analysis. For counting of γH2AX positive cells from FFPE samples, manual counting of at least 100 cells per section was performed. Positive cells are defined as those cells that form at least 5 foci per nucleus. Fluorescence images were acquired using a Nikon Eclipse TI inverted epifluorescence microscope equipped with a Nikon DS-Qi2 camera.

#### RAD51 and BRCA1 c-terminus score

Cell lines were cultured in 25cm plates. At 85% confluence, cells were trypsinised, centrifuged and washed twice with PBS. Cell pellets of 5x10^6^ cells were fixed for 24 h in 4% formaldehyde and then changed to 70% ethanol. Subsequently, cell pellets were embedded in paraffin. Sections were used to perform immunofluorescence, as explained above, with anti-RAD51 (Abcam, Rabbit polyclonal, catalog no. ab133534, 1:1,000), anti-BRCA1 (Santa Cruz, Mouse monoclonal, D-9, sc-6954, 1:50), anti-geminin (Rabbit polyclonal, ProteinTech Group, 10802-1-AP, 1:400 or Mouse monoclonal, NovoCastra NCL-L, 6096384, 1:100). Functional HRD was assessed by the RAD51 and BRCA1 c-terminus scores as the percentage of geminin-positive cells with 5 or more nuclear foci. One hundred geminin-positive cells from at least three representative areas of each sample were analyzed. The predefined cut-off of 10% was used to characterize HRR functionality. The scoring was performed by trained observers of Violeta Serra’s laboratory onto life images using a 60x immersion oil lens Nikon Eclipse TI inverted epifluorescence microscope.

#### Digital spatial profiling

FFPE HR-altered metastatic breast cancer biopsies (*n* = 12) collected before PARP inhibitor treatment (Figure S6A) were sectioned and stained for PANCK to identify tumor cells. Sample preparation and processing was performed by Citogen (Zaragoza, Spain). It followed Nanostring’s standard protocol, with 6–9 polygonal regions of interest (500–600 μm per sample) selected and processed using the NanoString DSP instrument. GeoMx digital spatial profiling (DSP) data were analyzed using the GeoMxTools R package (v. 3.10.0). Quality control, preprocessing and analysis were performed based on the pipeline from the GeoMX DSP Bioconductor’s workflow (https://bioconductor.org/packages/release/workflows/vignettes/GeoMxWorkflows/inst/doc/GeomxTools_RNA-NGS_Analysis.html). Data was normalised using quartile 3 normalization. For differential gene expression analysis between patient cohorts grouped by therapy response, linear mixed-effect model (LMM) were employed, as recommended by the Bioconductor’s pipeline. Pre-ranked (GSEA) was conducted using clusterProfiler (v. 4.14.4) with Hallmark gene sets from the MSigDB. The ranked list of genes was generated by multiplying the -log(*p*-value) by the sign of the logFC for each gene, based on the statistics obtained from the DE analysis.

### Quantification and statistical analysis

Analyses were performed using GraphPad Prism 9.0. Sample sizes were determined by power analysis. Data normal distribution was confirmed using Shapiro-Wilk test. Comparisons used two-tailed t-tests, one-way ANOVA with Tukey’s post-hoc test or two-way ANOVA with Sidak’s multiple comparisons test. *p* < 0.05 was considered significant. Data represent mean ± standard deviation of three independent experiments unless specified otherwise.

## Supplemental information

Document S1. Figures S1–S6

Document S2. Article plus supplemental information

## References

[1] Pelengaris S., Khan M., Evan G. (2002). c-MYC: more than just a matter of life and death. Nat. Rev. Cancer.

[2] Dang C.V. (2012). MYC on the path to cancer. Cell.

[3] Llombart V., Mansour M.R. (2022). Therapeutic targeting of “undruggable” MYC. EBioMedicine.

[4] Campaner S., Amati B. (2012). Two sides of the Myc-induced DNA damage response: from tumor suppression to tumor maintenance. Cell Div..

[5] Herold S., Herkert B., Eilers M. (2009). Facilitating replication under stress: an oncogenic function of MYC?. Nat. Rev. Cancer.

[6] Luoto K.R., Meng A.X., Wasylishen A.R., Zhao H., Coackley C.L., Penn L.Z., Bristow R.G. (2010). Tumor cell kill by c-MYC depletion: role of MYC-regulated genes that control DNA double-strand break repair. Cancer Res..

[7] Dominguez-Sola D., Gautier J. (2014). MYC and the control of DNA replication. Cold Spring Harb. Perspect. Med..

[8] Duffy M.J., O'Grady S., Tang M., Crown J. (2021). MYC as a target for cancer treatment. Cancer Treat Rev..

[9] Annibali D., Whitfield J.R., Favuzzi E., Jauset T., Serrano E., Cuartas I., Redondo-Campos S., Folch G., Gonzàlez-Juncà A., Sodir N.M. (2014). Myc inhibition is effective against glioma and reveals a role for Myc in proficient mitosis. Nat. Commun..

[10] Beaulieu M.E., Jauset T., Masso-Valles D., Martinez-Martin S., Rahl P., Maltais L., Zacarias-Fluck M.F., Casacuberta-Serra S., Serrano Del Pozo E., Fiore C. (2019). Intrinsic cell-penetrating activity propels Omomyc from proof of concept to viable anti-MYC therapy. Sci. Transl. Med..

[11] Masso-Valles D., Beaulieu M.E., Jauset T., Giuntini F., Zacarias-Fluck M.F., Foradada L., Martinez-Martin S., Serrano E., Martin-Fernandez G., Casacuberta-Serra S. (2022). MYC Inhibition Halts Metastatic Breast Cancer Progression by Blocking Growth, Invasion, and Seeding. Cancer Res. Commun..

[12] Soucek L., Whitfield J.R., Sodir N.M., Massó-Vallés D., Serrano E., Karnezis A.N., Swigart L.B., Evan G.I. (2013). Inhibition of Myc family proteins eradicates KRas-driven lung cancer in mice. Genes Dev..

[13] Zacarias-Fluck M.F., Masso-Valles D., Giuntini F., Gonzalez-Larreategui I., Kaur J., Casacuberta-Serra S., Jauset T., Martinez-Martin S., Martin-Fernandez G., Serrano Del Pozo E. (2023). Reducing MYC's transcriptional footprint unveils a good prognostic gene signature in melanoma. Genes Dev..

[14] Soucek L., Nasi S., Evan G.I. (2004). Omomyc expression in skin prevents Myc-induced papillomatosis. Cell Death Differ..

[15] Soucek L., Whitfield J., Martins C.P., Finch A.J., Murphy D.J., Sodir N.M., Karnezis A.N., Swigart L.B., Nasi S., Evan G.I. (2008). Modelling Myc inhibition as a cancer therapy. Nature.

[16] Garralda E., Beaulieu M.E., Moreno V., Casacuberta-Serra S., Martínez-Martín S., Foradada L., Alonso G., Massó-Vallés D., López-Estévez S., Jauset T. (2024). MYC targeting by OMO-103 in solid tumors: a phase 1 trial. Nat. Med..

[17] Xu J., Chen Y., Olopade O.I. (2010). MYC and Breast Cancer. Genes Cancer.

[18] Foulkes W.D., Smith I.E., Reis-Filho J.S. (2010). Triple-negative breast cancer. N. Engl. J. Med..

[19] Cortes J., Rugo H.S., Cescon D.W., Im S.A., Yusof M.M., Gallardo C., Lipatov O., Barrios C.H., Perez-Garcia J., Iwata H. (2022). Pembrolizumab plus Chemotherapy in Advanced Triple-Negative Breast Cancer. N. Engl. J. Med..

[20] Bardia A., Rugo H.S., Tolaney S.M., Loirat D., Punie K., Oliveira M., Brufsky A., Kalinsky K., Cortés J., Shaughnessy J.O. (2024). Final Results From the Randomized Phase III ASCENT Clinical Trial in Metastatic Triple-Negative Breast Cancer and Association of Outcomes by Human Epidermal Growth Factor Receptor 2 and Trophoblast Cell Surface Antigen 2 Expression. J. Clin. Oncol..

[21] Modi S., Jacot W., Yamashita T., Sohn J., Vidal M., Tokunaga E., Tsurutani J., Ueno N.T., Prat A., Chae Y.S. (2022). Trastuzumab Deruxtecan in Previously Treated HER2-Low Advanced Breast Cancer. N. Engl. J. Med..

[22] Fong P.C., Boss D.S., Yap T.A., Tutt A., Wu P., Mergui-Roelvink M., Mortimer P., Swaisland H., Lau A., O'Connor M.J. (2009). Inhibition of poly(ADP-ribose) polymerase in tumors from BRCA mutation carriers. N. Engl. J. Med..

[23] Lord C.J., Ashworth A. (2013). Mechanisms of resistance to therapies targeting BRCA-mutant cancers. Nat. Med..

[24] Rogakou E.P., Pilch D.R., Orr A.H., Ivanova V.S., Bonner W.M. (1998). DNA double-stranded breaks induce histone H2AX phosphorylation on serine 139. J. Biol. Chem..

[25] Graeser M., McCarthy A., Lord C.J., Savage K., Hills M., Salter J., Orr N., Parton M., Smith I.E., Reis-Filho J.S. (2010). A marker of homologous recombination predicts pathologic complete response to neoadjuvant chemotherapy in primary breast cancer. Clin. Cancer Res..

[26] Pellegrino B., Herencia-Ropero A., Llop-Guevara A., Pedretti F., Moles-Fernández A., Viaplana C., Villacampa G., Guzmán M., Rodríguez O., Grueso J. (2022). Preclinical In Vivo Validation of the RAD51 Test for Identification of Homologous Recombination-Deficient Tumors and Patient Stratification. Cancer Res..

[27] Rogakou E.P., Nieves-Neira W., Boon C., Pommier Y., Bonner W.M. (2000). Initiation of DNA fragmentation during apoptosis induces phosphorylation of H2AX histone at serine 139. J. Biol. Chem..

[28] Bonner W.M., Redon C.E., Dickey J.S., Nakamura A.J., Sedelnikova O.A., Solier S., Pommier Y. (2008). GammaH2AX and cancer. Nat. Rev. Cancer.

[29] Soucek L., Jucker R., Panacchia L., Ricordy R., Tatò F., Nasi S. (2002). Omomyc, a potential Myc dominant negative, enhances Myc-induced apoptosis. Cancer Res..

[30] Pedretti F., Abdalfttah M., Pellegrino B., Mateo F., Martínez-Sanz P., Herencia-Ropero A., Òdena A., Clavell-Revelles P., Casali G., Domènech H. (2025). Harnessing STING Signaling and Natural Killer Cells Overcomes PARP Inhibitor Resistance in Homologous Recombination-Deficient Breast Cancer. Cancer Res..

[31] Bretones G., Delgado M.D., León J. (2015). Myc and cell cycle control. Biochim. Biophys. Acta.

[32] Zhu J., Blenis J., Yuan J. (2008). Activation of PI3K/Akt and MAPK pathways regulates Myc-mediated transcription by phosphorylating and promoting the degradation of Mad1. Proc. Natl. Acad. Sci. USA.

[33] Wall M., Poortinga G., Hannan K.M., Pearson R.B., Hannan R.D., McArthur G.A. (2008). Translational control of c-MYC by rapamycin promotes terminal myeloid differentiation. Blood.

[34] Pocsfalvi G., Votta G., De Vincenzo A., Fiume I., Raj D.A.A., Marra G., Stoppelli M.P., Iaccarino I. (2011). Analysis of secretome changes uncovers an autocrine/paracrine component in the modulation of cell proliferation and motility by c-Myc. J. Proteome Res..

[35] Ciccia A., Elledge S.J. (2010). The DNA damage response: making it safe to play with knives. Mol. Cell.

[36] Jackson S.P., Bartek J. (2009). The DNA-damage response in human biology and disease. Nature.

[37] Lord C.J., Ashworth A. (2012). The DNA damage response and cancer therapy. Nature.

[38] Kuchenbaecker K.B., Hopper J.L., Barnes D.R., Phillips K.A., Mooij T.M., Roos-Blom M.J., Jervis S., van Leeuwen F.E., Milne R.L., Andrieu N. (2017). Risks of Breast, Ovarian, and Contralateral Breast Cancer for BRCA1 and BRCA2 Mutation Carriers. JAMA.

[39] Abramson V.G., Lehmann B.D., Ballinger T.J., Pietenpol J.A. (2015). Subtyping of triple-negative breast cancer: implications for therapy. Cancer.

[40] Noordermeer S.M., van Attikum H. (2019). PARP Inhibitor Resistance: A Tug-of-War in BRCA-Mutated Cells. Trends Cell Biol..

[41] Janysek D.C., Kim J., Duijf P.H.G., Dray E. (2021). Clinical use and mechanisms of resistance for PARP inhibitors in homologous recombination-deficient cancers. Transl. Oncol..

[42] Whitfield J.R., Beaulieu M.E., Soucek L. (2017). Strategies to Inhibit Myc and Their Clinical Applicability. Front. Cell Dev. Biol..

[43] Cancer Genome Atlas Network (2012). Comprehensive molecular portraits of human breast tumours. Nature.

[44] Gibson B.A., Kraus W.L. (2012). New insights into the molecular and cellular functions of poly(ADP-ribose) and PARPs. Nat. Rev. Mol. Cell Biol..

[45] Sung P., Krejci L., Van Komen S., Sehorn M.G. (2003). Rad51 recombinase and recombination mediators. J. Biol. Chem..

[46] Doha Z.O., Sears R.C. (2023). Unraveling MYC's Role in Orchestrating Tumor Intrinsic and Tumor Microenvironment Interactions Driving Tumorigenesis and Drug Resistance. Pathophysiology.

[47] Dang C.V. (2013). MYC, metabolism, cell growth, and tumorigenesis. Cold Spring Harb. Perspect. Med..

[48] Morrish F., Hockenbery D. (2014). MYC and mitochondrial biogenesis. Cold Spring Harb. Perspect. Med..

[49] Porro A., Iraci N., Soverini S., Diolaiti D., Gherardi S., Terragna C., Durante S., Valli E., Kalebic T., Bernardoni R. (2011). c-MYC oncoprotein dictates transcriptional profiles of ATP-binding cassette transporter genes in chronic myelogenous leukemia CD34+ hematopoietic progenitor cells. Mol. Cancer Res..

[50] Rottenberg S., Jaspers J.E., Kersbergen A., van der Burg E., Nygren A.O.H., Zander S.A.L., Derksen P.W.B., de Bruin M., Zevenhoven J., Lau A. (2008). High sensitivity of BRCA1-deficient mammary tumors to the PARP inhibitor AZD2281 alone and in combination with platinum drugs. Proc. Natl. Acad. Sci. USA.

[51] Gogola E., Duarte A.A., de Ruiter J.R., Wiegant W.W., Schmid J.A., de Bruijn R., James D.I., Guerrero Llobet S., Vis D.J., Annunziato S. (2018). Selective Loss of PARG Restores PARylation and Counteracts PARP Inhibitor-Mediated Synthetic Lethality. Cancer Cell.

[52] Chiou S.H., Jiang B.H., Yu Y.L., Chou S.J., Tsai P.H., Chang W.C., Chen L.K., Chen L.H., Chien Y., Chiou G.Y. (2013). Poly(ADP-ribose) polymerase 1 regulates nuclear reprogramming and promotes iPSC generation without c-Myc. J. Exp. Med..

[53] Cruz C., Castroviejo-Bermejo M., Gutiérrez-Enríquez S., Llop-Guevara A., Ibrahim Y.H., Gris-Oliver A., Bonache S., Morancho B., Bruna A., Rueda O.M. (2018). RAD51 foci as a functional biomarker of homologous recombination repair and PARP inhibitor resistance in germline BRCA-mutated breast cancer. Ann. Oncol..

[54] Kondrashova O., Nguyen M., Shield-Artin K., Tinker A.V., Teng N.N.H., Harrell M.I., Kuiper M.J., Ho G.Y., Barker H., Jasin M. (2017). Secondary Somatic Mutations Restoring RAD51C and RAD51D Associated with Acquired Resistance to the PARP Inhibitor Rucaparib in High-Grade Ovarian Carcinoma. Cancer Discov..

[55] Lheureux S., Bruce J.P., Burnier J.V., Karakasis K., Shaw P.A., Clarke B.A., Yang S.Y.C., Quevedo R., Li T., Dowar M. (2017). Somatic BRCA1/2 Recovery as a Resistance Mechanism After Exceptional Response to Poly (ADP-ribose) Polymerase Inhibition. J. Clin. Oncol..

[56] Das S.K., Kuzin V., Cameron D.P., Sanford S., Jha R.K., Nie Z., Rosello M.T., Holewinski R., Andresson T., Wisniewski J. (2022). MYC assembles and stimulates topoisomerases 1 and 2 in a “topoisome”. Mol. Cell.

[57] Yadav B., Wennerberg K., Aittokallio T., Tang J. (2015). Searching for Drug Synergy in Complex Dose-Response Landscapes Using an Interaction Potency Model. Comput. Struct. Biotechnol. J..

