# Supplementary material for: MYC inhibition by Omomyc causes DNA damage and overcomes PARPi resistance in breast cancer
Source: Cell Rep. 2025 Nov 21;44(12):116604. doi: 10.1016/j.celrep.2025.116604 (PMC12727680; doi:10.1016/j.celrep.2025.116604)
Supplement: Document S1. Figures S1–S6 [file mmc1.pdf]

Cell Reports, Volume *44*

Supplemental information

MYC inhibition by Omomyc causes DNA damage and overcomes PARPi resistance in breast cancer

Fabio Giuntini, Íñigo González-Larreategui, Andrea Herencia-Ropero, Silvia Casacuberta-Serra, Mariano F. Zacarías-Fluck, Magdalena Arnal, Flaminia Pedretti, Sandra Martínez-Martín, Hugo Thabussot, Virginia Castillo Cano, Judit Grueso, Laia Foradada, Erika Serrano, Sergio López-Estévez, Olga Rodriguez, Marta Guzman, Adela Rodriguez-Hernandez, Fara Brasó-Maristany, Alba Llop-Guevara, Judith Balmaña, Lara Nonell, Aleix Prat, Violeta Serra, Marie-Eve Beaulieu, Jonathan R. Whitfield, Daniel Massó-Vallés, and Laura Soucek

Supplementary Figure 1

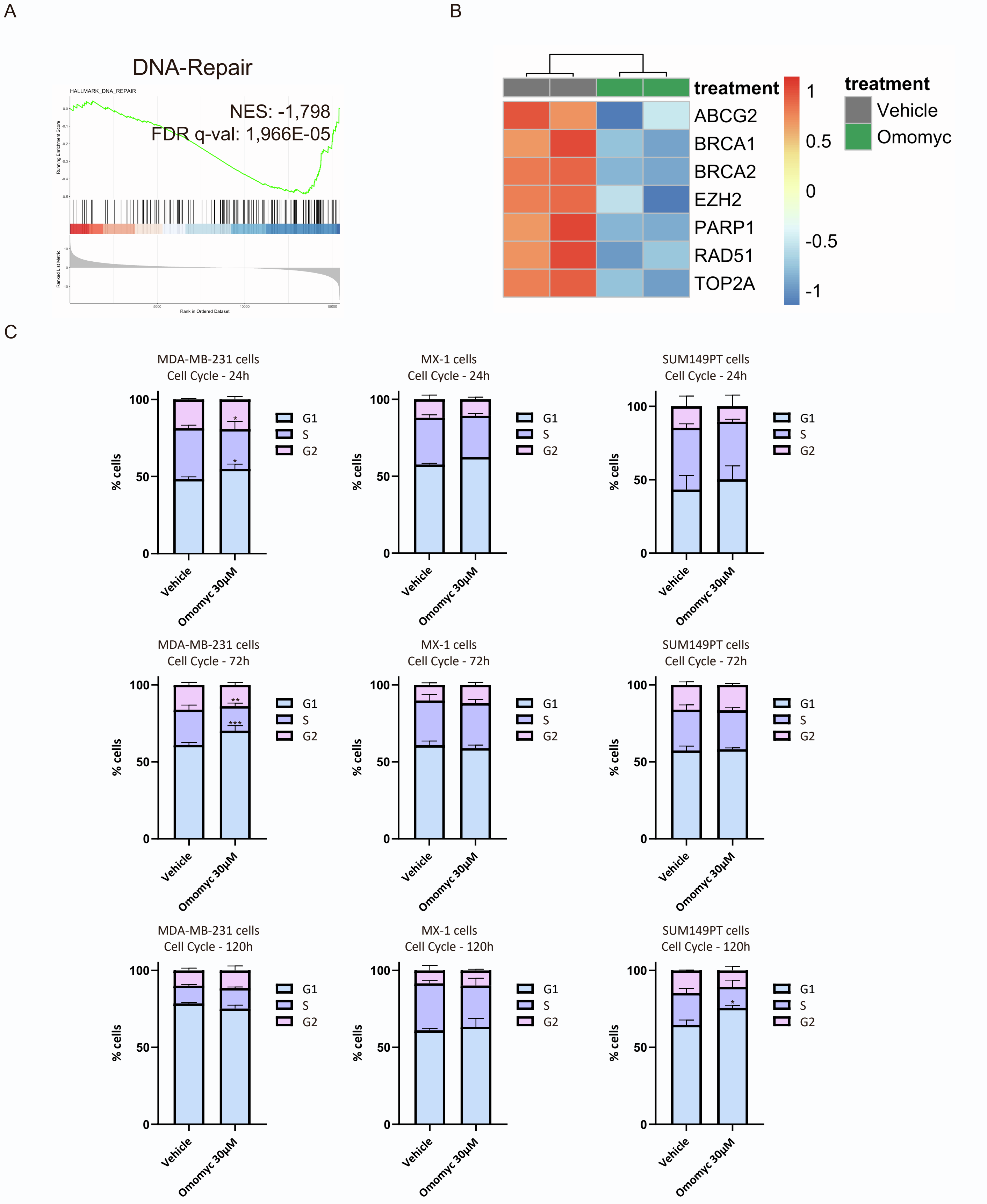

Supplementary Figure 1. Omomyc shuts down DDR pathways and induces DNA damage. Related to Figure 1. A) RNA sequencing analysis of Hallmark DNA repair gene set and **B)** Genes related to DDR differentially regulated upon Omomyc treatment in MDA-MB-231 cells as compared to vehicle-treated cells after 120 hours. **C)** Stacked bar-charts representing cell cycle phases (G1, S and G2 phase, see colour legend) in MDA-MB-231, MX-1 and SUM149PT cells (left to right) at 24, 72 and 120 hours (top to bottom). Asterisks represent statistical significance using ordinary two-way ANOVA test with Sidak's multiple comparisons test performed with GraphPad PRISM 9. *, $P < 0.05$; **, $P < 0.01$; ***, $P < 0.001$; error bars, mean ± SD.

Supplementary Figure 2

A

Cell Line	BRCA status	Olaparib GI50 (µM)	Talazoparib GI50 (µM)	RAD51 score (%)	BRCA1 (c-term) score (%)	PARPi sensitivity
SUM1315MO2	MUT	2.59	0.0034	1	1.5	Sensitive
**MDA-MB-231**	WT	6.43	0.034	16	45.8	**Resistant**
**MX-1**	MUT	70.31	15	11	27.5	**Resistant**
**SUM149PT**	MUT	13.89	0.04	17	19	**Resistant**

B

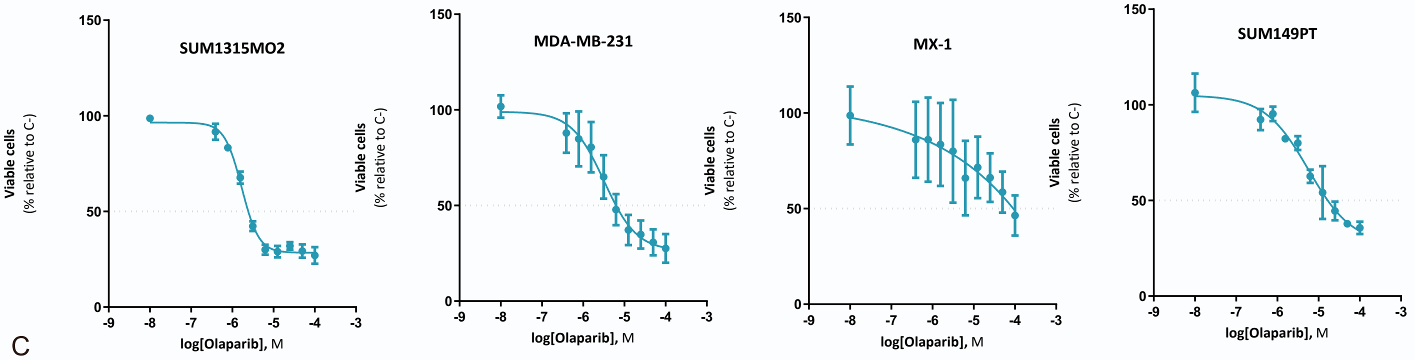

C

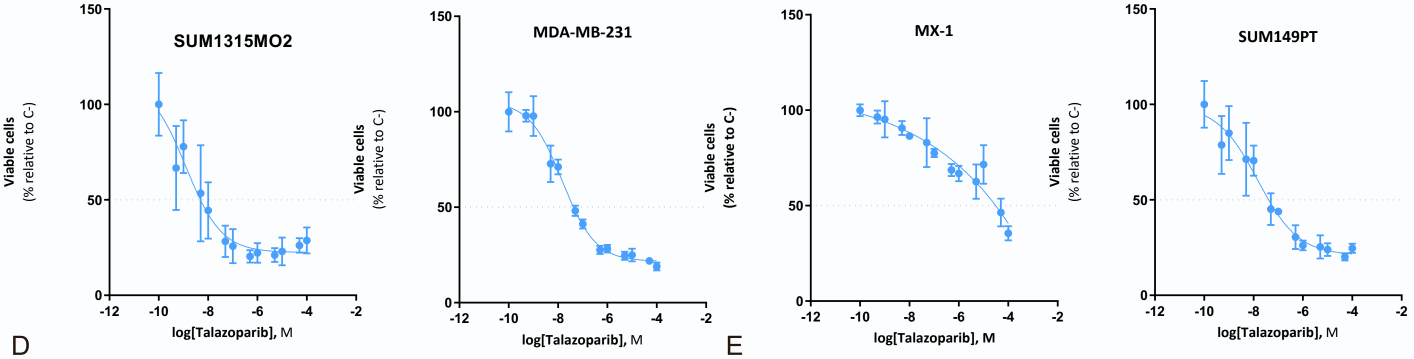

D

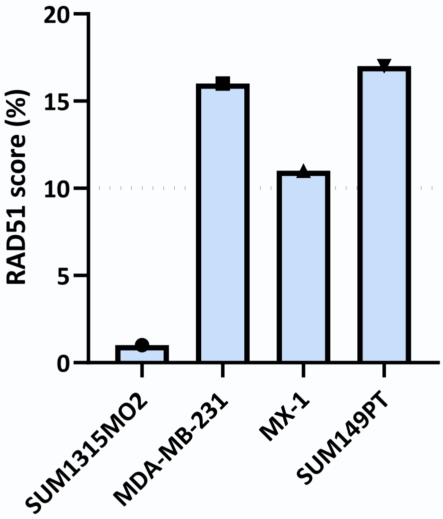

E

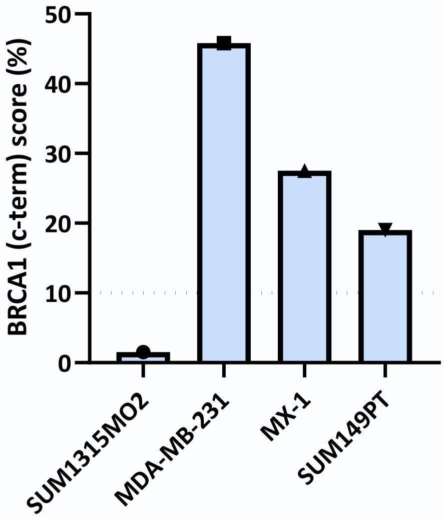

Supplementary Figure 2. Characterisation of a panel of TNBC PARPi-resistant cell lines. Related to Figure 1. A) Summary table with cell line, BRCA status, Olaparib GI50, Talazoparib GI50, RAD51 score, BRCA1 score and PARPi sensitivity details. **B)** Olaparib GI50 curves of SUM1315MO2, MDA-MB-231, MX-1 and SUM149PT (from left to right) treated with increasing concentration of the drug for 120 hours. Molar Log values are represented on X axis, while percentage of viable cells relative to vehicle-treated cells is depicted on Y axis. **C)** Talazoparib GI50 curves of SUM1315MO2, MDA-MB-231, MX-1 and SUM149PT (from left to right) treated with increasing concentration of the drug for 120 hours. Molar Log values are represented on X axis, while percentage of viable cells relative to vehicle treated cells is depicted on Y axis. **D)** Graphical representation of RAD51 score (%, Y axis), and **E)** BRCA1 (%, Y axis), where more than 10% is considered "high" while less than 10% is considered a "low" score, comparing different TNBC cells (X axis).

Supplementary Figure 3

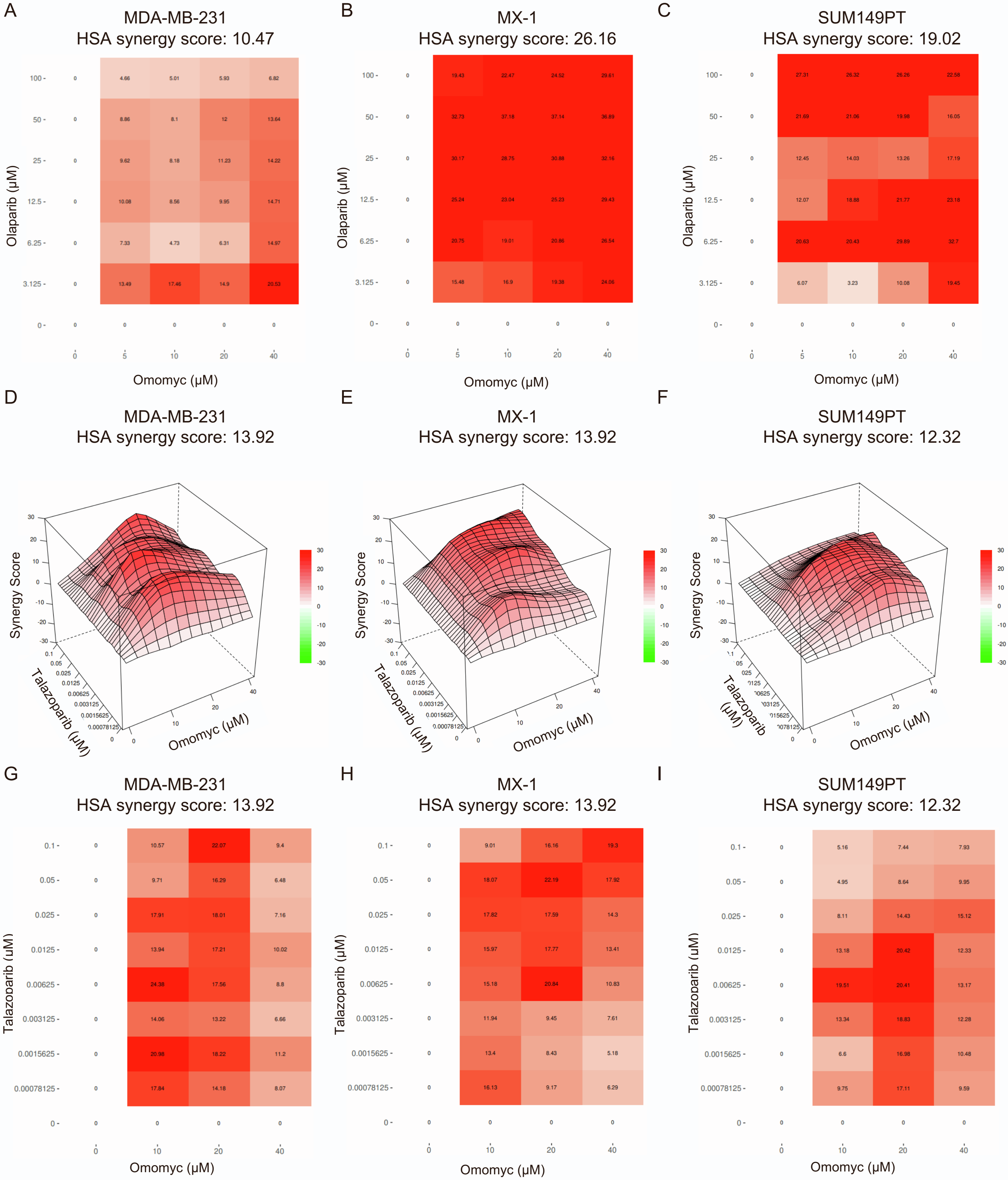

Supplementary Figure 3. Omomyc synergises with PARPi and enhances DNA damage in PARPi-resistant TNBC cells. Related to Figure 2. A) Matrix showing the HSA synergy score of each combination of Omomyc and Olaparib tested in MDA-MB-231, **B)** MX-1, and **C)** SUM149PT cells. **D)** Synergy plots depicting Gaddum's non-interaction model (HSA) for MDA-MB-231, **E)** MX-1, **F)** SUM149PT cells treated with increasing concentrations of Omomyc and/or Talazoparib. **G)** Matrix showing the HSA synergy score of each combination of Omomyc and Talazoparib tested in MDA-MB-231, **H)** MX-1, and **I)** SUM149PT cells.

Supplementary Figure 4

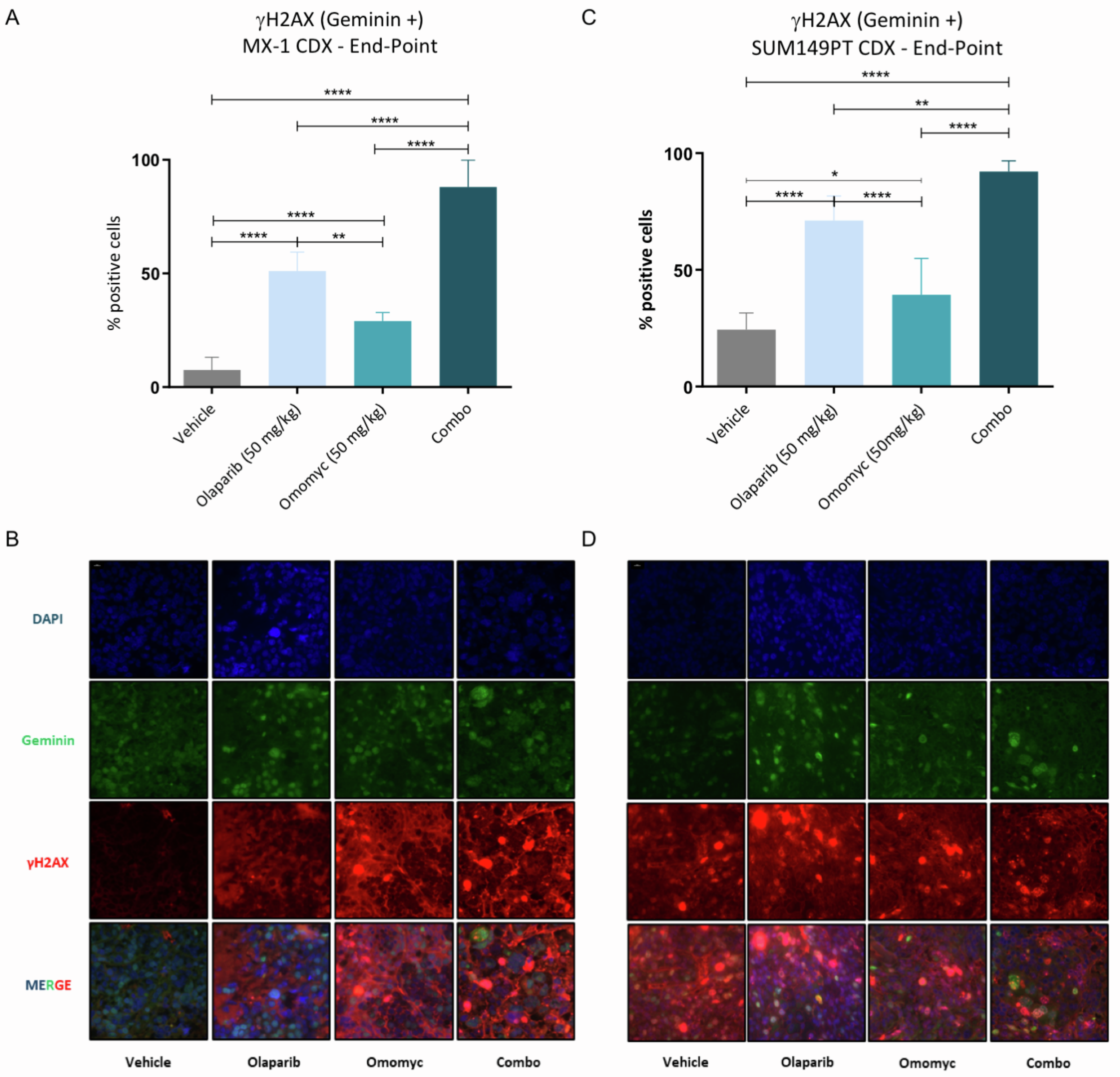

Supplementary Figure 4. Omomyc cooperates with Olaparib to induce durable antitumour response and DNA damage. Related to Figure 4. A) Bar-chart representing the percentage of cells positive for both γH2AX and geminin (Y axis) in each treatment group (X axis) in MX-1 CDX model. Asterisks represent statistical significance using of ordinary one-way ANOVA test with Tukey's multiple comparisons test performed with GraphPad PRISM 9. *, $P < 0.05$; **, $P < 0.01$; ***, $P < 0.001$; error bars, mean ± SD. **B)** Representative fluorescent microscopy images of FFPE cut show the differences in γH2AX in geminin-positive cells observed at end-point in MX-1 CDX mice. Scale 10μm. **C)** Bar-chart representing the percentage of cells positive for both γH2AX and geminin (Y axis) in each treatment group (X axis) in SUM149PT CDX model. Asterisks represent statistical significance using of ordinary one-way ANOVA test with Tukey's multiple comparisons test performed with GraphPad PRISM 9. *, $P < 0.05$; **, $P < 0.01$; ***, $P < 0.001$; error bars, mean ± SD. **D)** Representative fluorescent microscopy images of FFPE cut show the differences in γH2AX in geminin-positive cells observed at end-point in SUM149PT CDX mice. Scale 10μm.

Supplementary Figure 5

A

PDX ID	Clinical Subtype	Tumour Origin	HR Status	*Gene Altered*	BOR to Olaparib	Olaparib Sensistivity
**PDX093**	TNBC	Primary	Altered	*PALB2*	CR	Sensitive
**PDX093OR6**	TNBC	Primary	Altered	*PALB2*	PD	Resistant
**PDX124**	TNBC	Metastatic	Altered	*BRCA1*	SD	Sensitive
**PDX124OR3**	TNBC	Metastatic	Altered	*BRCA1*	PD	Resistant
**PDX127**	TNBC	Metastatic	Altered	*BRCA1*	PD	Resistant
**PDX173**	ER+/HER2+	Primary	Altered	*BRCA2*	CR	Sensitive
**PDX179**	TNBC	Metastatic	Altered	*BRCA1*	PD	Resistant
**PDX197**	TNBC	Primary	Altered	*BRCA1met*	CR	Sensitive
**PDX201STG**	ER+/HER2-	Metastatic	Altered	*BRCA1met*	CR	Sensitive
**PDX201ORSTG**	ER+/HER2-	Metastatic	WT (history HRD)		PD	Resistant
**PDX221**	TNBC	Primary	Altered	*BRCA1*	PD	Resistant
**PDX230**	TNBC	Primary	Altered	*BRCA1*	CR	Sensitive
**PDX230OR2**	TNBC	Primary	Altered	*BRCA1*	PD	Resistant
**PDX230OR8**	TNBC	Primary	NA	*NA*	PD	Resistant
**PDX236**	TNBC	Metastatic	Altered	*BRCA1*	PD	Resistant
**PDX252**	TNBC	Metastatic	Altered	*BRCA1*	PD	Resistant
**PDX270**	TNBC	Primary	Altered	*BRCA1met*	PD	Resistant
**PDX274**	TNBC	Metastatic	Altered	*BRCA1*	PD	Resistant
**PDX302**	TNBC	Metastatic	Altered	*BRCA1met*	PR	Sensitive
**PDX302OR1**	TNBC	Metastatic	WT (history HRD)		PD	Resistant
**PDX316STG**	TNBC	Primary	Altered	*BRCA1*	PD	Resistant
**PDX335**	TNBC	Metastatic	Altered	*BRCA2*	PD	Resistant
**PDX341**	TNBC	Metastatic	Altered	*BRCA1*	SD	Resistant
**PDX377**	TNBC	Metastatic	Altered	*BRCA1*	CR	Sensitive
**PDX377OR**	TNBC	Metastatic	Altered	*BRCA1*	PD	Resistant
**PDX384**	TNBC	Metastatic	Altered	*BRCA1*	SD	Sensitive
**PDX405OR**	TNBC	Metastatic	Altered	*BRCA2*	PD	Resistant
**PDX405T**	TNBC	Metastatic	Altered	*BRCA2*	PR	Sensitive
**PDX418**	ER+/HER2-	Metastatic	Altered	*BRCA1*	PD	Resistant
**PDX473**	TNBC	Primary	Altered	*BRCA1*	CR	Sensitive
**PDX474.1**	ER+/HER2-	Metastatic	Altered	*BRCA2*	PR	Sensitive
**PDX474.2**	ER+/HER2-	Metastatic	Altered	*BRCA2*	CR	Sensitive
**PDX474.2OR1**	ER+/HER2-	Metastatic	Altered	*BRCA2*	PD	Resistant
**PDX474.7**	ER+/HER2-	Metastatic	Altered	*BRCA2*	PD	Resistant
**PDX481**	ER+/HER2-	Metastatic	Altered	*BRCA2*	PR	Sensitive

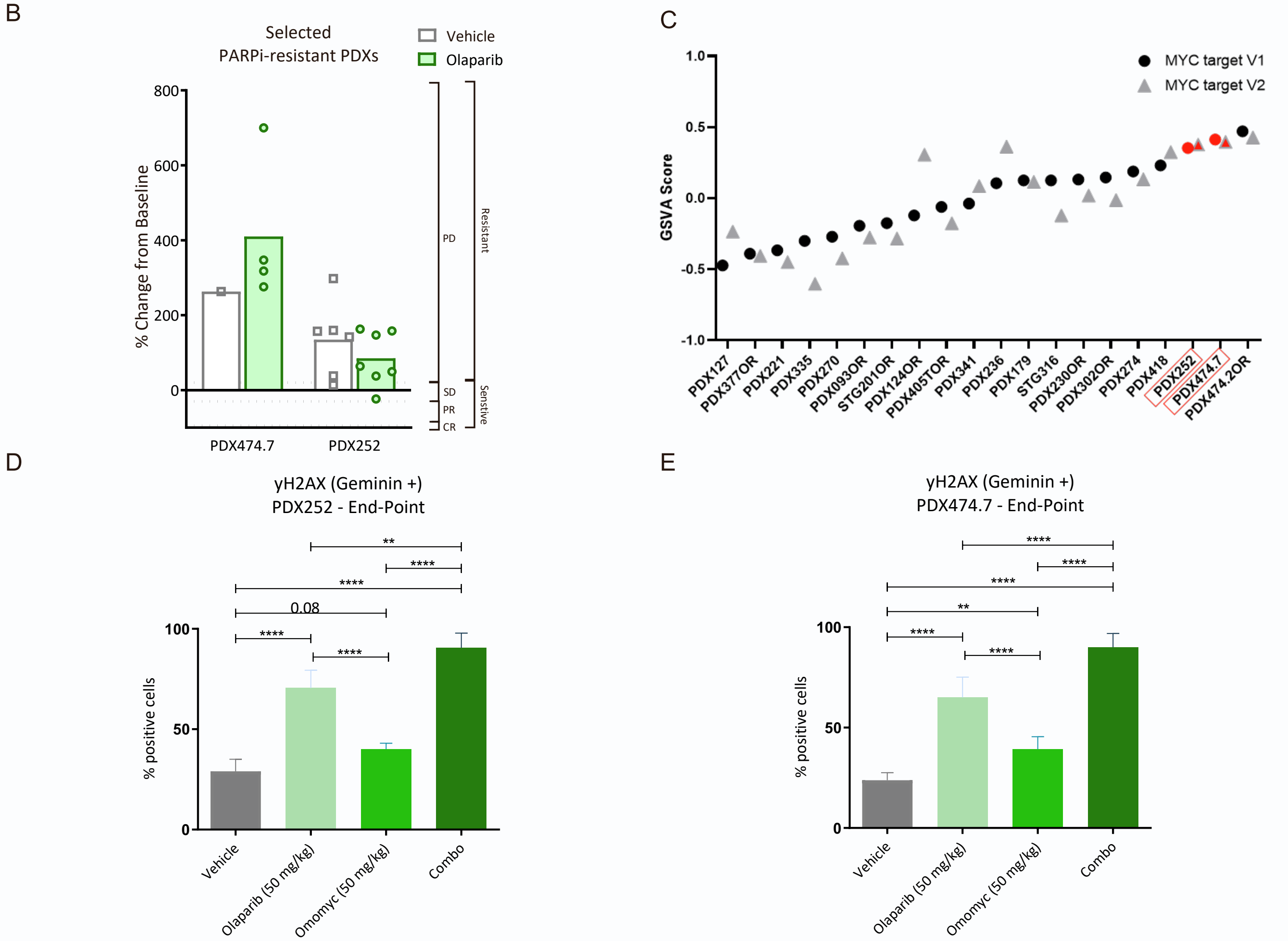

Supplementary Figure 5. Stronger MYC transcriptional signature correlates with resistance to Olaparib, which is reverted by Omomyc treatment. Related to Figure 5. A) Table describing a cohort of 35 HR-altered PDXs. Acronyms are used: homologous recombination (HR), best objective response (BOR), complete response (CR), partial response (PR), stable disease (SD) and progressive disease (PD). **B)** Paired bar-chart showing the average change from baseline (%) of PDX474.7 and PDX252 tumour growth when comparing vehicle-treated (white) or Olaparib 100mg/kg treated mice (light green) after three weeks. White squares (vehicle) and green circles (Olaparib) represent individual mice. Adapted from Pedretti et al., 2025, in press, Cancer Research. **C)** Gene-set Variation Analysis (GSVA) representing the enrichment of each resistant PDXs for MYC Target V1 and or V2. Models are ordered from less expressing to more expressing. **D)** Bar-chart representing the percentage of cells positive for both γH2AX and geminin (Y axis) in each treatment group (X axis) in the PDX252 model. Asterisks represent statistical significance using of ordinary one-way ANOVA test with Tukey's multiple comparisons test performed with GraphPad PRISM 9. *, $P < 0.05$; **, $P < 0.01$; ***, $P < 0.001$; error bars, mean ± SD. **E)** Bar-chart representing the percentage of cells positive for both γH2AX and geminin (Y axis) in each treatment group (X axis) in the PDX474.7 model. Asterisks represent statistical significance using of ordinary one-way ANOVA test with Tukey's multiple comparisons test performed with GraphPad PRISM 9. *, $P < 0.05$; **, $P < 0.01$; ***, $P < 0.001$; error bars, mean ± SD.

Supplementary Figure 6

A

Sample ID	Tissue	PARPi	BOR	HR Mutation
**HCB 1.3**	Breast	Talazoparib	PD	*BRCA1*
**HCB 1.4**	Bone	Talazopraib	PD	*BRCA2*
**HCB 1.10**	Breast	Olaparib	PD	*BRCA1*
**HCB 1.14**	Bone	Talazoparib	CR	*BRCA2*
**HCB 1.16**	Breast	Olaparib	PD	*BRCA1*
**HCB 1.19**	Breast	Olaparib	PD	WT
**HCB 1.23**	Breast	Olaparib	PD	*BRCA2*
**VHIO 466**	Lymph Node	Olaparib	CR	*BRCA2*
**VHIO 493**	Breast	Talazoparib	PR	*BRCA2*
**VHIO 498**	Breast	Olaparib	PR	*BRCA2*
**VHIO 560**	Lymph node	Olaparib	PR	*BRCA1*
**VHIO 570**	Brain	Olaparib	CR	*BRCA2*

B

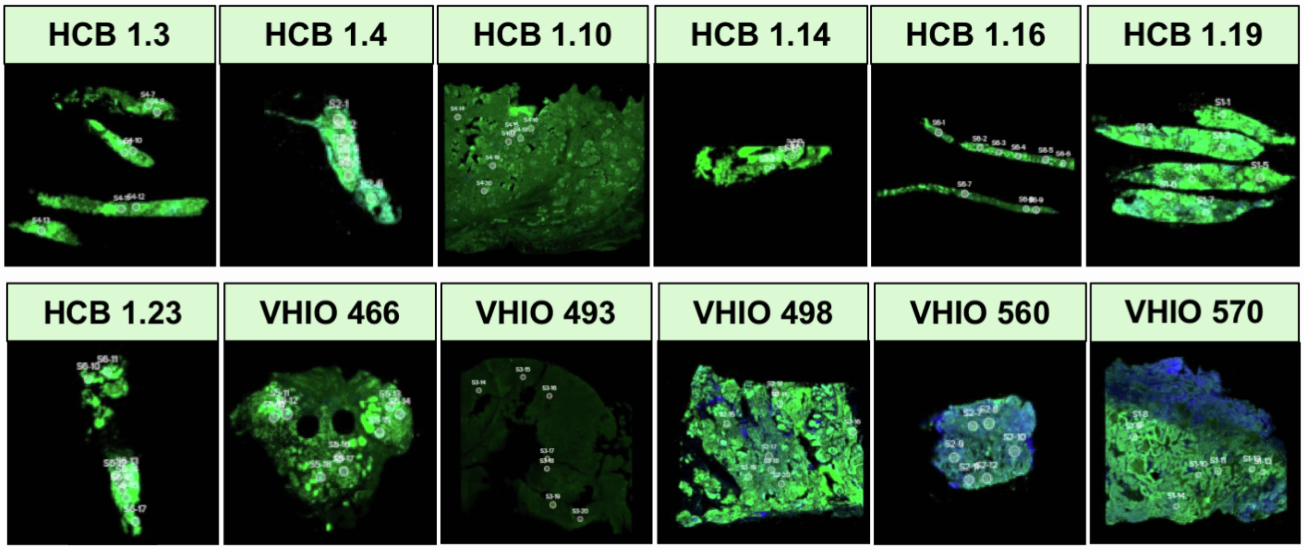

C

PD patients

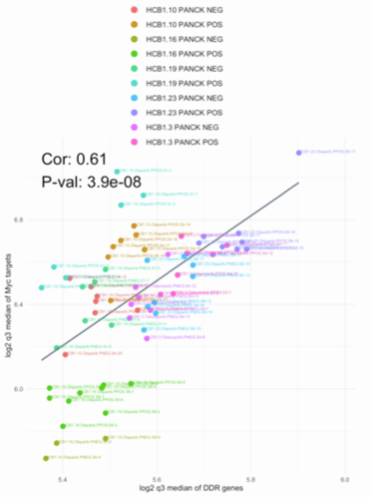

D

PR patients

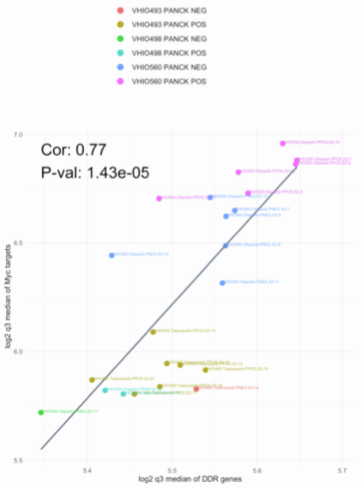

E

CR patients

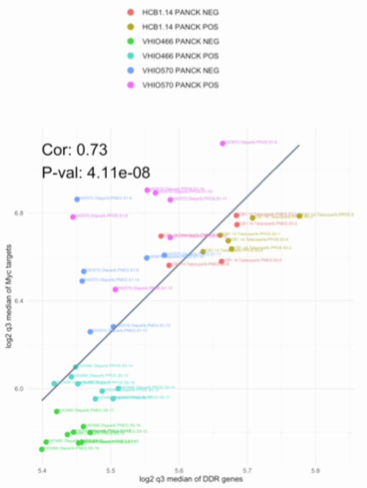

F

PD vs PR+CR
In PANCK-

MYC targets V1

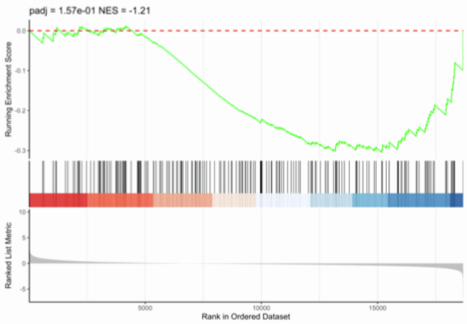

G

PD vs PR+CR
In PANCK-

DNA Repair

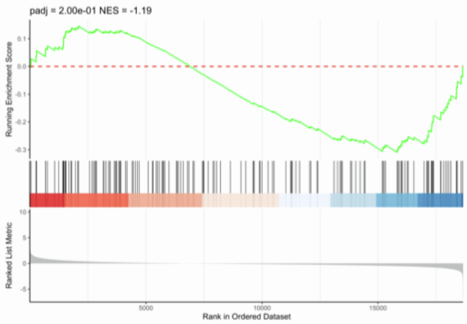

Supplementary Figure 6: MYC transcriptional signature can serve as a predictor of PARPi response. Related to Figure 6. **A)** Table describing the cohort of 12 patients' biopsies used for DSP. **B)** Images of each biopsy used for DSP analysis and the selected region of interest (ROI) for each of them. **C)** Correlation analysis performed in PANCK+ and PANCK- regions of each biopsy in progressive disease (PD) patients, **(D)** partial response (PR) patients and **(E)** complete response (CR) patients. **F)** GSEA comparing MYC targets V1 gene-set in PD as compared to PR+CR in PANCK- regions. **G)** GSEA comparing DNA repair gene-set in PD as compared to PR+CR in PANCK- regions.
